# Efficacy and safety/tolerability of antipsychotics in the treatment of adult patients with major depressive disorder: a systematic review and meta-analysis

**DOI:** 10.1017/S0033291722000745

**Published:** 2023-07

**Authors:** Taishiro Kishimoto, Katsuhiko Hagi, Shunya Kurokawa, John M. Kane, Christoph U. Correll

**Affiliations:** 1 Department of Neuropsychiatry, Keio University School of Medicine, Tokyo, Japan; 2 Department of Psychiatry, The Zucker Hillside Hospital, Northwell Health, Glen Oaks, New York, USA; 3 Department of Psychiatry and Molecular Medicine, Donald and Barbara Zucker School of Medicine at Hofstra/Northwell, Hempstead, New York, USA; 4 The Feinstein Institute for Medical Research, Center for Psychiatric Neuroscience, Manhasset, New York, USA; 5 Sumitomo Dainippon Pharma Co, Ltd, Medical Affairs, Tokyo, Japan; 6 Department of Child and Adolescent Psychiatry, Charité Universitätsmedizin, Berlin, Germany

**Keywords:** Adjunctive therapy, antipsychotics, major depressive disorder, meta-analysis, monotherapy

## Abstract

**Background:**

Antipsychotics are widely used in the treatment of major depressive disorder (MDD), but there has been no comprehensive meta-analytic assessment that examined their use as monotherapy and adjunctive therapy.

**Methods:**

A systematic review and a meta-analysis were conducted on randomized placebo-controlled trials (RCTs) that reported on the efficacy and safety/tolerability of antipsychotics for the treatment of adults with MDD. Data of both monotherapy and adjunctive antipsychotic use were extracted, but analyzed separately using a random-effects model. Co-primary outcomes were study-defined-treatment response and intolerability-related discontinuation. We also illustrated the risk/benefit balance of antipsychotics for MDD, using two-dimensional graphs representing the primary efficacy and safety/tolerability outcome. Secondary outcomes included psychopathology, remission, all-cause-discontinuation, inefficacy-related discontinuation, and adverse events.

**Results:**

Forty-five RCTs with 12 724 patients were included in the analysis. In monotherapy (studies = 13, *n* = 4375), amisulpride [1.99 (1.55–2.55)], sulpiride [1.50 (1.03–2.17)], and quetiapine [1.48 (1.23–1.78)] were significantly superior to placebo regarding treatment response. However, intolerability-related discontinuations were significantly higher compared to placebo with amisulpride and quetiapine. In adjunctive therapy (studies = 32, *n* = 8349), ziprasidone [1.80 (1.07–3.04)], risperidone [1.59 (1.19–2.14)], aripiprazole [1.54 (1.35–1.76)], brexpiprazole [1.41 (1.21–1.66)], cariprazine [1.27 (1.07–1.52)], and quetiapine [1.23 (1.08–1.41)] were significantly superior to placebo regarding treatment response. However, of these antipsychotics that were superior to placebo, only risperidone was equivalent to placebo regarding discontinuation due to intolerability, while the other antipsychotics were inferior.

**Conclusion:**

Results suggest that there are significant differences regarding the risk/benefit ratio among antipsychotics for MDD, which should inform clinical care.

## Introduction

Major depressive disorder (MDD) is a prevalent mental disorder affecting 246–286 million people globally (GBD 2017 Disease and Injury Incidence and Prevalence Collaborators, 2018). It is a serious threat to physical and mental health, characterized by persistent depressed mood, anhedonia, feelings of worthless or guilt, loss of motivation, somatic symptoms, cognitive impairments, sleep dysfunction, as well as suicidal thoughts and/or attempts (Malhi & Mann, 2018; Rakel, 1999; Ribeiro, Huang, Fox, & Franklin, 2018; Shim, Noh, Yoon, Mun, & Hahm, 2019; World Health Organisation, 2018). Approximately 5–12% of males and 9–26% of females will suffer from at least one episode of MDD over their lifetime, and about 50% of patients will experience a second depressive episode (Crown et al., 2002; Finley, 2009; Kessler et al., 2003). The personal, societal, and economic burden of MDD is enormous. According to a World Health Organization report, MDD is projected to be one of the three leading causes of disease burden worldwide by the year of 2030, representing that it puts a heavy burden on the public healthcare systems and on the global economy. This impact is even greater when taking into account the fact that MDD is widely recognized major risk factor for other disabling conditions, such as substance abuse disorders (Swendsen et al., 2010), and cardiovascular diseases (Penninx, 2017).

Although antidepressants are efficacious treatments for depression, a substantial number of patients do not respond adequately to these drugs. The STAR*D study indicated that only approximately half of patients treated for MDD show a favorable treatment response to antidepressants and only about one-third achieve remission (Trivedi et al., 2006). These results highlight the need for other agents to alleviate the symptoms of MDD. While antidepressants presumably normalize serotonin and norepinephrine, several lines of evidence suggest that antipsychotics, which regulate serotonin, noradrenalin as well as dopamine in different ways, may also play an important role in the treatment of MDD (Montgomery, 2008; Nutt, 2006).

Antipsychotics are widely used in the treatment of MDD. In the United States in 2007 and 2008, there were an estimated 3.9 million treatment visits per year in which an antipsychotic was prescribed for depression, and almost all of these (96%) involved the prescription of a second-generation antipsychotic (SGA) drug (Alexander, Gallagher, Mascola, Moloney, & Stafford, 2011).

Currently, four SGAs – aripiprazole, brexpiprazole, olanzapine, and quetiapine – have received approval from the US Food and Drug Administration (FDA) as adjunctive therapies in adults with MDD, while none have been approved as monotherapy.

In clinical practice, however, controversy exists as to the optimal selection of a particular antipsychotic for the treatment of patients with MDD, as SGAs differ in their selectivity for 5-HT receptors and/or affinity and intrinsic activity regarding D2 receptors as well as their effects on different brain regions (Blier & Szabo, 2005).

Although the efficacy and tolerability of adjunctive SGA therapy in treatment-resistant and suboptimally responsive depression have been summarized in previous meta-analyses of randomized controlled trials (RCTs) (Komossa, Depping, Gaudchau, Kissling, & Leucht, 2010; Nelson & Papakostas, 2009; Papakostas, Shelton, Smith, & Fava, 2007; Spielmans et al., 2013), there has been no comprehensive evaluation of efficacy and safety/tolerability separating the clinically incompatible antipsychotic monotherapy and adjunctive strategies.

Therefore, it is highly important to have a broader view of a wide range of evidence, considering the strength/weakness of each treatment strategy, in order to thoroughly examine the effectiveness of antipsychotics for MDD. To the best of our knowledge, this is the largest meta-analysis examining the efficacy and safety/tolerability of antipsychotic treatment for depression both as monotherapy and as adjunctive therapy.

## Methods

### Search strategy and selection criteria

This meta-analysis followed PRISMA guidelines for reporting meta-analyses of RCTs (Moher, Liberati, Tetzlaff, & Altman, 2009). This study protocol was registered with open science frame (https://osf.io/dashboard). We selected placebo-controlled RCTs of antipsychotics for adults with MDD as per DSM or ICD criteria. However, we excluded trials of patients with psychotic depression or mixed features in depression because their pathophysiology and treatment are different from those of MDD, which was the target of most trials. We conducted a systematic literature search without language restrictions, using MEDLINE/PubMed, Cochrane library, Scopus, and Embase from database inception (last search: 6/16/2021), for RCTs of patients with MDD. We also searched for unpublished studies, such as conference proceedings and clinical trial registries (http://clinicaltrials.gov/). Search terms included synonyms of (1) MDD AND (2) antipsychotics AND (3) controlled AND (4) randomized AND (5) clinical trial. Hand searches of reference lists of relevant publications were also conducted.

### Data extraction

Data were extracted independently by two or more reviewers (KH, SK, TK) experienced in conducting literature searches and data extraction for meta-analyses. Disagreements were resolved by consensus. Data extraction was double-checked by two or three investigators (KH, SK, TK).

The primary efficacy outcome was study-defined treatment response (in most of the cases percentage of patients with ⩾50% improvement in depressive symptom scale scores from baseline), with few studies defining response as ‘very much’ or ‘much improved’ according to the clinical Global Impressions-improvement scale (CGI-I). The primary safety/tolerability outcome was treatment intolerability-related discontinuation.

Secondary outcomes included: (i) depressive symptom scale scores measured by the Montgomery–Åsberg Depression Rating Scale (MADRS), the Hamilton Depression Scale (HAM-D), or other standardized observer-rated scales for depression at endpoint. When studies provided multiple depressive symptom scale scores, we used the MADRS as the depressive symptom measure, as it was the most commonly reported scale; (ii) global illness severity (CGI-Severity = CGI-S); (iii) suicide-related outcomes such as suicidal ideation and suicidal attempt; (iv) anxiety symptoms, measured by the Hamilton Anxiety Scale (HAM-A); (v) remission, defined by a score of ⩽7 on the 17-item HAM-D (⩽8 for all other longer versions of the HAM-D), or ⩽10 on the MADRS; (vi) study discontinuation due to any reason; (vii) inefficacy-related discontinuation; (viii) laboratory parameters; and (ix) adverse events. All eligible trials were assessed for their methodological quality using the Cochrane Collaboration's tool for assessing risk of bias (‘The Cochrane Collaboration. Cochrane handbook for systematic reviews of interventions (Version 5.1.0)’, 2011). We extracted data on study design, patient, illness, and treatment characteristics.

### Statistical analysis

All data were double-entered into and meta-analyzed with Comprehensive Meta-Analysis Version 3 (BioStat; Englewood, New Jersey) using a random-effects model, as heterogeneity among studies was expected (DerSimonian & Laird, 1986). Whenever a study involved ⩾2 appropriate dose groups of an antipsychotic, the dose arms were pooled for the main analysis and considered as one. Dichotomous outcomes were expressed as the pooled relative risk (RR), and continuous outcomes were expressed as standardized mean difference (SMD) using the inverse variance method. RR and SMD values for individual antipsychotics and administration-type (monotherapy or adjunctive therapy) were reported with their 95% confidence intervals (CIs). RR values >1 indicate superiority of the antipsychotic for positive outcomes, such as treatment response and remission, while RR values <1 indicate superiority of the antipsychotic for negative outcomes, such as intolerability-related discontinuation, discontinuation due to inefficacy, all-cause discontinuation, and incidence of an individual adverse event. For simplicity, we adjusted effect sizes, so that SMDs <0 indicate the superiority of antipsychotic treatments, independent of whether a lower or higher value represents a positive outcome in the original scale. In the primary analyses, antipsychotics and placebo were compared at the study endpoint.

We also assessed clinical benefit and harm of treatments for MDD, using the number-needed-to-treat (NNT) for benefits, and the number-needed-to-harm (NNH), for harms, which were calculated with the pooled mean frequency of a beneficial or harmful outcome, respectively, whenever the RR for that outcome was statistically significant. In order to visualize the risk/benefit balance of antipsychotics for MDD, we illustrated two-dimensional graphs with the *x*-axis representing the RR for treatment response and the *y*-axis representing the RR for intolerability-related discontinuations. We explored study heterogeneity using the χ^2^ test of homogeneity and *I*^2^ statistics, with *p* < 0.05 and *I*^2^ > 50%, respectively, indicating significant heterogeneity. All analyses were two-tailed with *α* = 0.05. As is customary in meta-analyses, no adjustments were made to *p* values for multiple comparisons; therefore, the *p* values should be interpreted with caution.

We conducted *a priori*-defined subgroup and meta-regression analyses for the co-primary outcomes, seeking to identify potential moderators, mediators, methodological biases and whether the findings extended to clinically relevant sub-populations and treatment groups. The subgroup characteristics were: (1) sponsorship (industry/academia), (2) country, (3) location (international/USA/Europe/Asia/rest of the world), (4) publication year (1999 or earlier/2000–2009/2010 or later), (5) antipsychotic drug category (first-generation antipsychotic (FGA), SGA), (6) study quality (high risk of bias yes/no), (7) concealment (open/single-blinded, double-blinded), (8) psychotic depression (including/excluding patients with psychotic depression), and (9) antipsychotic dose [using five dose groups based on the daily prescribed dose divided by defined daily dose (DDD) as per WHO (Methodology); i.e. 0 to ⩽0.25 daily dose ratio; 0.25 to ⩽0.5 daily dose ratio, 0.5 to ⩽0.75 daily dose ratio, 0.75 to <1 daily dose ratio; ⩾1.0 daily dose ratio]. The moderator or mediator variables were (1) age, (2) percent males, (3) percent Caucasian, (4) publication year, (5) sample size, (6) trial duration, (7) illness duration, (8) number of lifetime depressive episodes, (9) number of depressive episodes in the past year.

Risk of bias was evaluated in accordance with the Cochrane Handbook for Systematic Reviews of interventions, using the following parameters: adequacy of sequence generation; allocation concealment; blinding of participants, personnel and outcome assessors; incomplete outcome data; and selective outcome reporting. Publication bias was assessed by visually inspecting funnel plots. Additionally, we calculated the Egger regression test (Egger, Davey Smith, Schneider, & Minder, 1997) for the co-primary outcomes whenever ⩾3 studies were analyzed. In case of apparent publication bias, we used the trim-and-fill method. Finally, we calculated the fail–safe number of negative studies that would be required to nullify (i.e. make *p* > 0.05) the statistically significant effect size.

## Results

The initial search produced 4377 records, and 4334 records were excluded on title/abstract level. Of the remaining 43 records, five were excluded after full-text review and seven studies were added through hand-search of relevant reviews, yielding 45 meta-analyzable studies. For references of included studies, see online Supplementary material (Fig. S1 and Table S2).

Among the 13 antipsychotic monotherapy studies, five (38.5%) had at least one area with high-risk of bias. Seven (53.8%) monotherapy studies had at least five areas with low-risk of bias, and three (23.1%) had low-risk of bias in all areas (online Supplementary Table S1).

Among the 32 adjunctive antipsychotic studies, eight (25.0%) had at least one area with high-risk of bias. Seventeen (53.1%) adjunctive studies had at least five areas with low-risk of bias, and nine (28.1%) had low-risk of bias in all areas (online Supplementary Table S1).

### Study, patient, and treatment characteristics

Studies were published between 1974 and 2019. Thirteen studies (*n* = 4375) were conducted as antipsychotic monotherapy. All of them were double-blind studies. Nine studies were sponsored by industry, three by academia, and in one study the sponsor was not reported. Six studies were conducted in the outpatient setting and in the remaining seven studies the treatment setting was not reported. The median number of participants was 310 (range = 22–776), and the mean duration was 12.2 (range = 1–52) weeks. The mean age of participants was 45.2 ± 7.6 years, 64.6% were female, and 76.7% were white. There were six antipsychotic–placebo pairs in monotherapy trials (amisulpride, fluphenazine, haloperidol, quetiapine, sulpiride, ziprasidone) (online Supplementary Table S2).

Thirty-two RCTs (*n* = 8349) were conducted with antipsychotic treatment adjunctive to antidepressants, 30 studies were double-blind, one each single-blind and open label. Thirty-one studies were sponsored by industry and one by academia. Nineteen studies were conducted in the outpatient setting, three in the inpatient setting, one study included both in- and outpatients, and in nine studies the treatment setting was not reported. The median number of participants was 203 (range = 20–819), and the mean duration was 7.4 (range = 2–36) weeks. The mean age of participants was 45.4 ± 5.2 years, 65.8% were female, and 82.1% were white.

There were 11 antipsychotic–placebo pairs in trials with adjunctive therapy (aripiprazole, brexpiprazole, cariprazine, iloperidone, olanzapine, oxypertine, pipamperone, quetiapine, risperidone, thioridazine, ziprasidone) (online Supplementary Table S2).

### Primary outcomes

#### Monotherapy

**Fig. 1.**
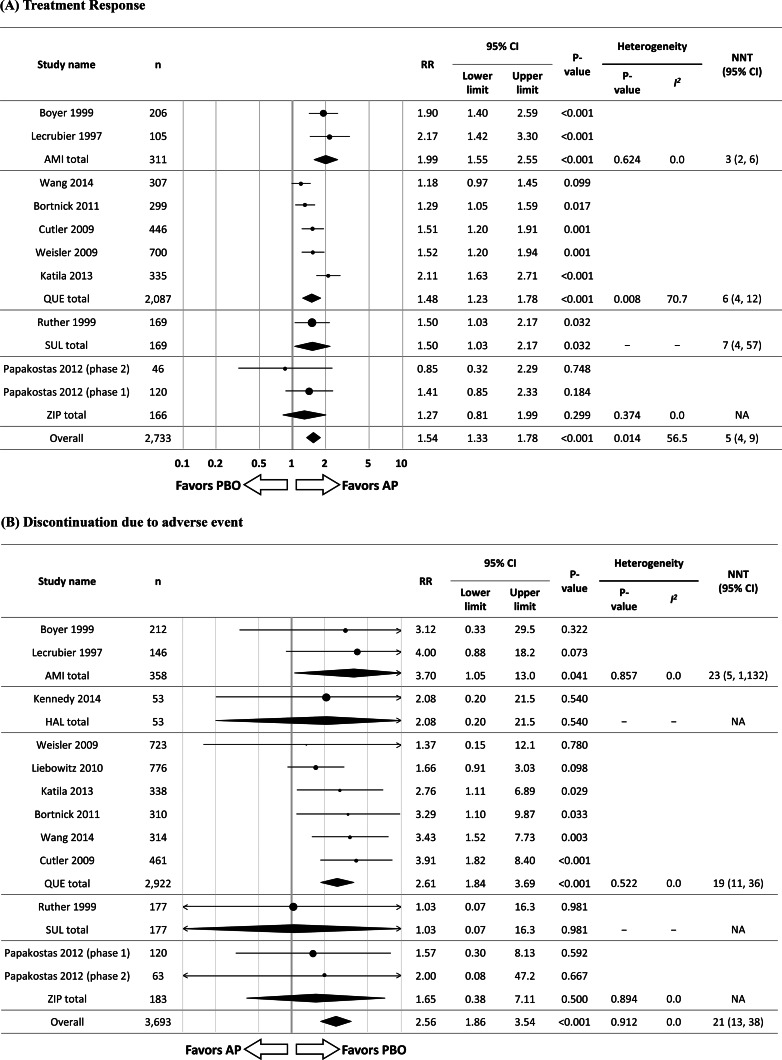
Antipsychotic drug monotherapy *v*. placebo. *Notes*: RR values >1 indicate superiority of antipsychotics compared to placebo for treatment response, while RR values >1 indicate inferiority of antipsychotics compared to placebo for discontinuation due to adverse event. NNTs for treatment response and NNHs for discontinuation due to adverse event were calculated. AMI, amisulpride; AP, antipsychotic drug; CI, confidence interval; HAL, haloperidol; *n*, number of patients; PBO, placebo; QUE, quetiapine; RR, risk ratio; SUL, sulpiride; ZIP, ziprasidone.

Overall, antipsychotics were significantly superior to placebo regarding treatment response (*N* = 10, *n* = 2733, RR = 1.54, 95% CI 1.33–1.78, *p* < 0.001; NNT = 5, 95% CI 4–9). Individually, amisulpride (*N* = 2, *n* = 311, RR = 1.99, 95% CI 1.55–2.55, *p* < 0.001; NNT = 3, 95% CI 2–6), sulpiride (*N* = 1, *n* = 169, RR = 1.50, 95% CI 1.03–2.17, *p* = 0.032; NNT = 7, 95% CI 4–57), and quetiapine (*N* = 5, *n* = 2087, RR = 1.48, 95% CI 1.23–1.78, *p* < 0.001; NNT = 6, 95% CI 4–12) were associated with significantly greater treatment response than placebo. Conversely, ziprasidone (*N* = 2, *n* = 166, RR = 1.27, 95% CI 0.81–1.99, *p* = 0.299) did not significantly separate from placebo regarding treatment response [Fig. 1(*a*)]. Three RCTs of olanzapine monotherapy, Shelton (2005) (*Journal of Clinical Psychiatry*, 2005; 66(10): 1289–1297), Weissman (2012) (*Psychiatry Research*, 2012; 197(3): 221–226), and Rothschild (2004) (*Journal of Clinical Psychopharmacology*, 200424(4): 365–373), have been published. The first two studies, Shelton (2005) and Weissman (2012), were excluded, as they did not have a placebo arm to evaluate the efficacy and safety/tolerability of olanzapine monotherapy. The third study, Rothschild (2004), was excluded, as this study reported the results of olanzapine monotherapy for MDD with psychotic features. Suppes (2016) (*American Journal of Psychiatry*, 2016; 173(4): 400–407) was excluded, as this study reported results from randomized placebo-controlled RCT on lurasidone monotherapy for MDD with mixed features where the effect size was large, which could have been due to greater responsiveness of mixed features than of depressive symptoms alone.

**Fig. 2.**
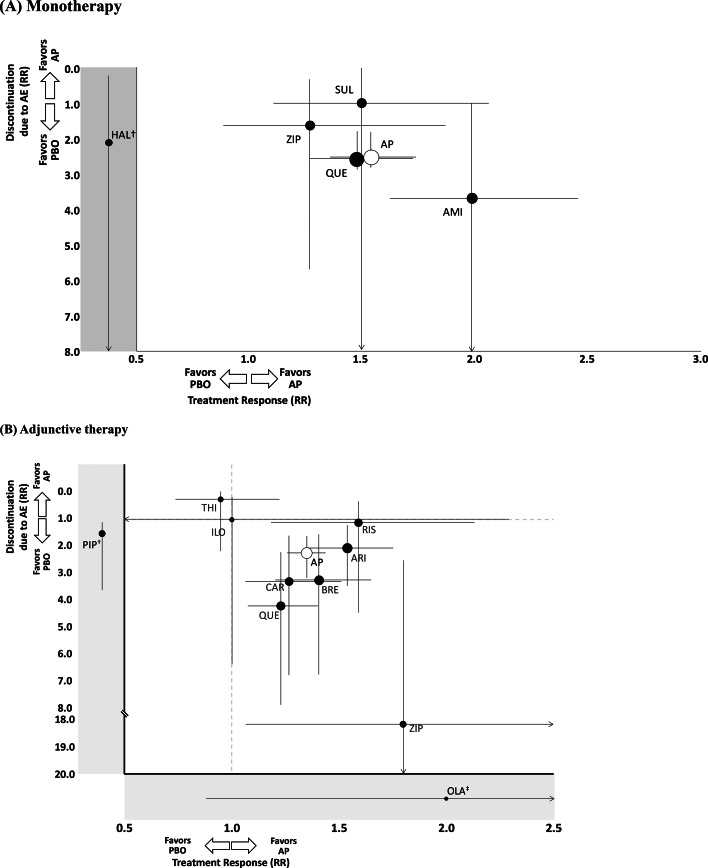
Two-dimensional graph about treatment response and discontinuation due to adverse event (a) monotherapy. *Notes*: Data are reported as RRs in comparison with placebo. Error bars are 95% CIs. Size of every circle is proportional to the logarithm of sample size. ^†^Treatment response was not reported. RRs for treatment response and for discontinuation due to adverse event are as follows: AP overall: RR = 1.54, CI 1.33–1.78, *p* < 0.001; RR = 2.56, CI 1.86–3.54, *p* < 0.001; AMI: RR = 1.99, CI 1.55–2.55, *p* < 0.001; RR = 3.70, CI 1.05–13.0, *p* = 0.041; HAL: treatment response not reported; RR = 2.08, CI 0.20–21.5, *p* = 0.540; QUE: RR = 1.48, CI 1.23–1.78, *p* < 0.001; RR = 2.61, CI 1.84–3.69, *p* < 0.001; SUL: RR = 1.50, CI 1.03–2.17, *p* = 0.032; RR = 1.03, CI 0.07–16.3, *p* = 0.981; ZIP: RR = 1.27, CI 0.81–1.99, *p* = 0.299; RR = 1.65, CI 0.38–7.11, *p* = 0.500. AE, adverse event; AMI, amisulpride; AP, antipsychotic drug; CI, confidence interval; HAL, haloperidol; PBO, placebo; QUE, quetiapine; RR, risk ratio; SUL, sulpiride; ZIP, ziprasidone. (b) Adjunctive therapy. *Notes*: Data are reported as RRs in comparison with placebo. Error bars are 95% CIs. Size of every circle is proportional to the logarithm of sample size. ^†^Treatment response was not reported. ^‡^Discontinuation due to adverse event was not reported. RRs for treatment response and for discontinuation due to adverse event are as follows: AP overall: RR = 1.35, CI 1.26–1.45, *p* < 0.001; RR = 2.39, CI 1.69–3.38, *p* < 0.001; ARI: RR = 1.54, CI 1.35–1.76, *p* < 0.001; RR = 2.08, CI 1.23–3.51, *p* = 0.006; BRE: RR = 1.41, CI 1.21–1.66, *p* < 0.001; RR = 3.24, CI 1.54–6.79, *p* = 0.002; CAR: RR = 1.27, CI 1.07–1.52, *p* = 0.007; RR = 3.30, CI 1.59–6.84, *p* = 0.001; ILO: RR = 1.00, CI 0.44–2.29, *p* = 1.000; RR = 1.00, 0.16–6.42, *p* = 1.000; OLA: RR = 2.00, CI 0.88–4.54, *p* = 0.098; discontinuation due to AE not reported. PIP: treatment response not reported; RR = 0.49, CI 0.09–2.62, *p* = 0.408; QUE: RR = 1.23, CI 1.08–1.41, *p* = 0.002; RR = 4.19, CI 2.22–7.90, *p* < 0.001; RIS: RR = 1.59, CI 1.19–2.14, *p* = 0.002; RR = 1.15, CI 0.29–4.53, *p* = 0.840; THI: RR = 0.95, CI 0.74–1.22, *p* = 0.696; RR = 0.26, CI 0.03–2.25, *p* = 0.223; ZIP: RR = 1.80, CI 1.07–3.04, *p* = 0.028; RR = 18.2, CI 2.53–131, *p* = 0.004. AE, adverse event; AP, antipsychotic drug; ARI, aripiprazole; BRE, brexpiprazole; CAR, cariprazine; CI, confidence interval; ILO, iloperidone; OLA, olanzapine; PBO, placebo; PIP, pipamperone; QUE, quetiapine; RIS, risperidone; RR, risk ratio; THI, thioridazine; ZIP, ziprasidone.

Overall, antipsychotic monotherapy was associated with significantly higher intolerability-related discontinuation (*N* = 12, *n* = 3693, RR = 2.56, 95% CI 1.86–3.54, *p* < 0.001; NNH = 21, 95% CI 13–38). Individually, amisulpride (*N* = 2, *n* = 358, RR = 3.70, 95% CI 1.05–13.0, *p* = 0.041; NNH = 23, 95% CI 5–1132) and quetiapine (*N* = 7, *n* = 2944, RR = 2.61, 95% CI 1.84–3.69, *p* < 0.001; NNH = 19, 95% CI 11–36) were associated with significantly higher intolerability-related discontinuation than placebo. Conversely, this was not the case for sulpiride (*N* = 1, *n* = 177, RR = 1.03, 95% CI 0.07–16.3, *p* = 0.981) and ziprasidone (*N* = 2, *n* = 183, RR = 1.65, 95% CI 0.38–7.11, *p* = 0.500) [Fig. 1(*b*)]. The risk/benefit balance of antipsychotic monotherapy for MDD is illustrated in Fig. 2(*a*) (*x*-axis indicates the RR for treatment response, *y*-axis indicates intolerability-related discontinuations). The summarized findings of this meta-analysis for antipsychotic monotherapy are presented in online Supplementary Table S3.

#### Adjunctive therapy

**Fig. 3.**
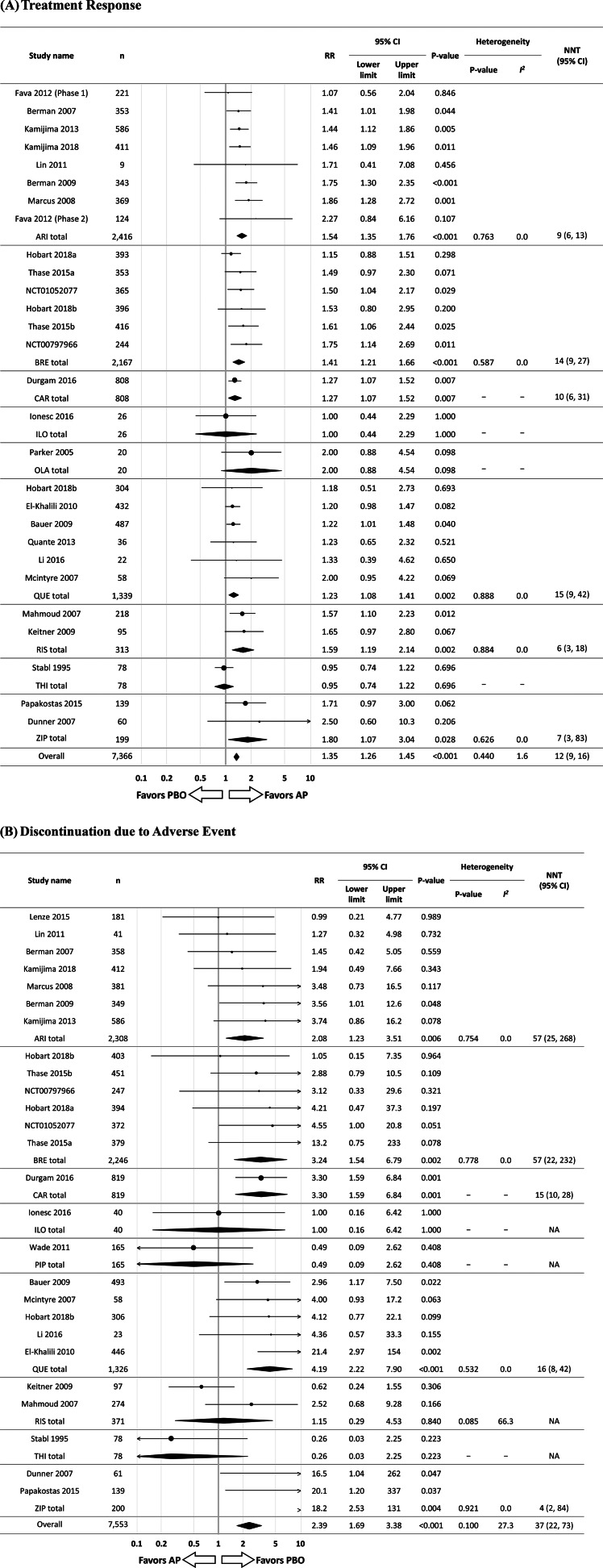
Antipsychotic drug adjunctive therapy *v*. placebo. *Notes*: RR values >1 indicate superiority of antipsychotics compared to placebo for treatment response, while RR values >1 indicate inferiority of antipsychotics compared to placebo for discontinuation due to adverse event. NNTs for treatment response and NNHs for discontinuation due to adverse event were calculated. AP, antipsychotic drug; ARI, aripiprazole; BRE, brexpiprazole; CAR, cariprazine; CI, confidence interval; ILO, iloperidone; *n*, number of patients; OLA, olanzapine; PBO, placebo; PIP, pipamperone; QUE, quetiapine; RIS, risperidone; RR, risk ratio; THI, thioridazine; ZIP, ziprasidone.

Overall, antipsychotics were significantly superior to placebo regarding treatment response (*N* = 28, *n* = 7366, RR = 1.35, 95% CI 1.26–1.45, *p* < 0.001; NNT = 12, 95% CI 9–16) [Fig. 3(a)]. Individually, ziprasidone (*N* = 2, *n* = 199, RR = 1.80, 95% CI 1.07–3.04, *p* = 0.028; NNT = 7, 95% CI 3–83), risperidone (*N* = 2, *n* = 313, RR = 1.59, 95% CI 1.19–2.14, *p* = 0.002; NNT = 6, 95% CI 3–18), aripiprazole (*N* = 8, *n* = 2416, RR = 1.54, 95% CI 1.35–1.76, *p* < 0.001; NNT = 9, 95% CI 6–13), brexpiprazole (*N* = 6, *n* = 2167, RR = 1.41, 95% CI 1.21–1.66, *p* < 0.001; NNT = 14, 95% CI 9–27), cariprazine (*N* = 1, *n* = 808, RR = 1.27, 95% CI 1.07–1.52, *p* = 0.007; NNT = 10, 95% CI 6–31), and quetiapine (*N* = 6, *n* = 1339, RR = 1.23, 95% CI 1.08–1.41, *p* = 0.002; NNT = 15, 95% CI 9–42) were significantly superior to placebo regarding treatment response. Conversely, iloperidone (*N* = 1, *n* = 26, RR = 1.00, 95% CI 0.44–2.29, *p* = 1.000), olanzapine (*N* = 1, *n* = 20, RR = 2.00, 95% CI 0.88–4.54, *p* = 0.098), perphenazine (*N* = 1, *n* = 30, RR = 1.14, 95% CI 0.53–2.45, *p* = 0.732), and thioridazine (*N* = 1, *n* = 78, RR = 0.95, 95% CI 0.74–1.22, *p* = 0.696) did not significantly separate from placebo.

Overall, adjunctive antipsychotic therapy was associated with significantly higher intolerability-related discontinuation than placebo (*N* = 26, *n* = 7553, RR = 2.39, 95% CI 1.69–3.38, *p* < 0.001; NNH = 37, 95% CI 27–73). Individually, ziprasidone (*N* = 2, *n* = 200, RR = 18.2, 95% CI 2.53–131, *p* = 0.004; NNH = 4, 95% CI 2–84), quetiapine (*N* = 5, *n* = 1326, RR = 4.19, 95% CI 2.22–7.90, *p* < 0.001; NNH = 16, 95% CI 8–42), cariprazine (*N* = 1, *n* = 819, RR = 3.30, 95% CI 1.59–6.84, *p* = 0.001; NNH = 15, 95% CI 10–28), brexpiprazole (*N* = 6, *n* = 2246, RR = 3.24, 95% CI 1.54–6.79, *p* = 0.002; NNH = 57, 95% CI 22–232), and aripiprazole (*N* = 7, *n* = 2308, RR = 2.08, 95% CI 1.23–3.51, *p* = 0.006; 95% CI 57, 95% CI 25–268) were associated with significantly higher intolerability-related discontinuation. Conversely, iloperidone (*N* = 1, *n* = 40, RR = 1.00, 95% CI 0.16–6.42, *p* = 1.000), pipamperone (*N* = 1, *n* = 165, RR = 0.49, 95% CI 0.09–2.62, *p* = 0.408), risperidone (*N* = 2, *n* = 371, RR = 1.15, 95% CI 0.29–4.53, *p* = 0.840), and thioridazine (*N* = 1, *n* = 78, RR = 0.26, 95% CI 0.03–2.25, *p* = 0.223) were not significantly different from placebo in intolerability-related discontinuation [Fig. 3(*b*)]. The risk/benefit balance of antipsychotic adjunctive therapy to antidepressants is illustrated in Fig. 2(*b*). The summarized findings of this and the largest prior meta-analysis for antipsychotic adjunctive therapy are presented in online Supplementary Table S3 in order to illustrate the added knowledge.

### Secondary outcomes

Table 1 and online Supplementary Table S4 summarize the meta-analytic results of secondary outcomes including all-cause discontinuation; discontinuation due to inefficacy; remission; depressive symptom scale score change; at least one adverse event; extrapyramidal symptom (EPS)-related adverse events; arousal-related adverse events; metabolic and endocrine adverse events; and suicide-related outcomes.

**Table 1.** Summary data table (secondary outcomes)

Category	Subcategory	Specific outcomes	Medication	*N*	*n*	RR/*SMD*	95% CI		Results: *p* value	Heterogeneity		NNT^a^ (95% CI)	NNH^a^ (95% CI)
							Lower limit	Upper limit		*p* value	*I*^2^		
***Monotherapy***													
Effectiveness	All cause discontinuation		AMI	2	358	0.79	0.58	1.07	0.127	0.523	0.0		NA
			HAL	1	53	2.77	0.82	9.31	0.100	−	−		NA
			QUE	7	2944	1.07	0.94	1.22	0.326	0.130	39.3		NA
			SUL	1	177	1.22	0.58	2.58	0.598	−	−		NA
			**ZIP**	**2**	**183**	**2.27**	**1.29**	**4.00**	**0.004**	0.493	0.0		**6 (3–23)**
Efficacy	Discontinuation due to lack of efficacy		**AMI**	**2**	**358**	**0.49**	**0.30**	**0.80**	**0.004**	0.923	0.0	**10**^b^ **(7–24)**	
			HAL	1	53	5.19	0.26	103	0.281	−	−		NA
			**QUE**	**6**	**2922**	**0.31**	**0.12**	**0.84**	**0.021**	0.117	43.2	**52**^b^ **(41–219)**	
			SUL	1	177	0.26	0.03	2.27	0.222	−	−		NA
			ZIP	2	183	1.87	0.39	8.97	0.433	0.358	0.0		NA
	Remission		**QUE**	**5**	**2087**	**1.46**	**1.08**	**1.99**	**0.016**	**0.003**	**75.0**	**10 (5–59)**	
			ZIP	2	166	1.18	0.67	2.08	0.560	0.234	29.5	NA	
	*Depressive symptom scale score*		***AMI***	***1***	***206***	***−0.68***	***−0.96***	***−0.39***	***<0.001***	*−*	*−*	*−*	*−*
			*HAL*	*1*	*53*	*0*.*01*	*−0*.*53*	*0*.*55*	*0*.*971*	*−*	*−*	*−*	*−*
			***QUE***	***4***	***1428***	***−0.40***	***−0.63***	***−0.18***	***<0.001***	***0.023***	***68.5***	*−*	*−*
			*ZIP*	*2*	*166*	*0*.*04*	*−0*.*57*	*0*.*65*	*0*.*899*	*0.087*	*66.0*	*−*	*−*
Safety	At least one AE		AMI	2	357	1.05	0.77	1.44	0.746	−	−		NA
			**QUE**	**6**	**2788**	**1.22**	**1.13**	**1.32**	**<0.001**	**0.034**	**58.6**		**8 (5–13)**
			**SUL**	**1**	**171**	**1.24**	**1.01**	**1.52**	**0.042**	−	−		**7 (4–122)**
	EPS-related AEs	Any EPS	**QUE**	**5**	**2135**	**1.75**	**1.11**	**2.74**	**0.015**	0.453	0.0		**44 (19–290)**
		Akathisia	QUE	2	360	1.06	0.23	4.96	0.939	0.398	0.0		NA
			ZIP	1	205	1.56	0.10	24.6	0.751	−	−		NA
		Restlessness	QUE	1	338	3.11	0.13	75.8	0.486	−	−		NA
			SUL	1	171	1.06	0.07	16.7	0.967	−	−		NA
		Tremor	QUE	1	338	1.04	0.07	16.4	0.980	−	−		NA
	Arousal-related AEs	Insomnia	AMI	1	212	0.45	0.12	1.68	0.231	−	−		NA
			QUE	7	2964	0.79	0.47	1.34	0.383	**0.001**	**72.3**		NA
			SUL	1	171	7.42	0.39	141	0.183	−	−		NA
			ZIP	1	205	1.04	0.18	6.10	0.964	−	−		NA
		Sedation	**QUE**	**7**	**3687**	**5.94**	**4.14**	**8.51**	**<0.001**	0.704	0.0		**11 (7–17)**
			**ZIP**	**1**	**205**	**6.77**	**1.99**	**23.0**	**0.002**	−	−		**8 (5–19)**
		Somnolence	**QUE**	**9**	**4047**	**4.22**	**2.86**	**6.21**	**<0.001**	**0.030**	**52.8**		**8 (5–13)**
	Metabolic and endocrine AEs	Weight increased	AMI	1	212	6.23	0.76	50.87	0.088	−	−		NA
			QUE	3	1452	2.28	0.80	6.50	0.124	**0.021**	**74.1**		NA
			SUL	1	171	1.06	0.07	16.7	0.967	−	−		NA
			ZIP	1	205	0.52	0.02	12.6	0.686	−	−		NA
		Increased appetite	**QUE**	**5**^c^	**2573**	**1.87**	**1.10**	**3.18**	**0.021**	0.379	2.6		**42 (17–371)**
			SUL	1	171	5.30	0.26	109	0.280	−	−		NA
			ZIP	1	205	10.9	0.57	208	0.113	−	−		NA
		⩾7% weight gain	**QUE**	**6**	**2838**	**2.08**	**1.17**	**3.69**	**0.012**	0.787	0.0		**68 (28–424)**
		*Body weight change*	***QUE***	***6***	***2882***	***0.26***	***0.11***	***0.42***	***0.001***	***0.002***	***73.7***	*−*	*−*
		*Glucose change*	*QUE*	*6*	*2731*	*0*.*06*	*−0*.*02*	*0*.*14*	*0*.*145*	*0.480*	*0.0*	*−*	*−*
		*Triglycerides change*	***QUE***	***6***	***2694***	***0.09***	***0.01***	***0.17***	***0.028***	*0.721*	*0.0*	*−*	*−*
		*Total cholesterol change*	*QUE*	*6*	*2694*	*0*.*04*	*−0*.*10*	*0*.*18*	*0*.*569*	***0.011***	***66.4***	*−*	*−*
		*HDL cholesterol change*	*QUE*	*6*	*2694*	*0*.*08*	*−0*.*04*	*0*.*19*	*0*.*186*	*0.092*	*47.2*	*−*	*−*
		*LDL cholesterol change*	*QUE*	*6*	*2694*	*0*.*00*	*−0*.*12*	*0*.*13*	*0*.*967*	***0.045***	***55.8***	*−*	*−*
		Sexual dysfunction	QUE	4	1797	1.06	0.40	2.81	0.909	0.563	0.0		NA
			ZIP	1	205	0.78	0.07	8.48	0.839	−	−		NA
		*Prolactin change*	*QUE*	*6*	*2786*	*0*.*03*	*−0*.*06*	*0*.*13*	*0*.*501*	*0.210*	*30.0*	*−*	*−*
	Suicide related outcomes	Suicidal attempt	QUE	4	1705	0.81	0.18	3.54	0.777	0.541	0.0		NA
		Suicidal ideation	QUE	1	312	2.96	0.12	72.2	0.505	−	−		NA
			ZIP	1	205	0.52	0.02	12.6	0.686	−	−		NA
***Adjunctive therapy***													
Effectiveness	All cause discontinuation		ARI	9	2671	1.10	0.89	1.35	0.387	0.624	0.0		NA
			**BRE**	**6**	**2246**	**1.37**	**1.05**	**1.80**	**0.021**	0.754	0.0		**37 (17–282)**
			**CAR**	**1**	**819**	**1.59**	**1.12**	**2.26**	**0.009**	−	−		**13 (8–41)**
			ILO	1	40	1.00	0.43	2.33	1.000	−	−		NA
			OLA	1	126	2.91	0.31	27.2	0.350	−	−		NA
			PIP	1	165	0.66	0.36	1.20	0.175	−	−		NA
			**QUE**	**6**	**1362**	**1.29**	**1.00**	**1.67**	**0.047**	0.347	10.7		**23 (10–2105)**
			RIS	2	297	0.77	0.13	4.61	0.774	0.078	67.8		NA
			THI	1	78	0.70	0.28	1.78	0.457	−	−		NA
			ZIP	2	200	1.59	1.00	2.53	0.051	0.456	0.0		NA
Efficacy	Discontinuation due to lack of efficacy		ARI	7	2308	0.84	0.33	2.15	0.720	0.718	0.0		NA
			BRE	6	1416	0.44	0.13	1.46	0.180	0.601	0.0		NA
			CAR	1	819	0.65	0.15	2.89	0.574	−	−		NA
			ILO	1	40	NA	NA	NA	NA	−	−		NA
			PIP	1	165	2.96	0.12	71.72	0.504	−	−		NA
			**QUE**	**5**	**1326**	**0.26**	**0.09**	**0.71**	**0.008**	0.327	13.6	**32**^b^ **(26–79)**	
			RIS	1	274	1.13	0.35	3.62	0.835	−	−		NA
			ZIP	2	200	2.87	0.31	26.95	0.355	−	−		NA
	Remission		**ARI**	**9**	**2597**	**1.59**	**1.37**	**1.85**	**<0.001**	0.890	0.0	**10 (7–16)**	
			**BRE**	**6**	**2167**	**1.42**	**1.17**	**1.72**	**<0.001**	0.589	0.0	**19 (11–46)**	
			CAR	1	808	1.07	0.86	1.33	0.556	−	−	NA	
			ILO	1	26	1.00	0.32	3.17	1.000	−	−	NA	
			QUE	7	1453	1.25	0.89	1.76	0.202	**0.032**	**56.6**	NA	
			**RIS**	**2**	**313**	**2.21**	**1.41**	**3.47**	**0.001**	0.875	0.0	**6 (3–18)**	
			**ZIP**	**2**	**199**	**2.35**	**1.10**	**5.02**	**0.027**	0.949	0.0	**9 (3–111)**	
	*Depressive symptom scale score*		***ARI***	***9***	***2586***	***−0.34***	***−0.43***	***−0.25***	***<0.001***	*0.255*	*21.2*	*−*	*−*
			***BRE***	***6***	***2167***	***−0.26***	***−0.34***	***−0.18***	***<0.001***	*0.765*	*0.0*	*−*	*−*
			***CAR***	***1***	***808***	***−0.17***	***−0.32***	***−0.02***	***0.023***	*−*	*−*	*−*	*−*
			*ILO*	*1*	*26*	*−0*.*08*	*−0*.*85*	*0*.*69*	*0*.*843*	*−*	*−*	*−*	*−*
			*OLA*	*1*	*20*	*−0*.*35*	*−1*.*23*	*0*.*53*	*0*.*436*	*−*	*−*	*−*	*−*
			*PER*	*1*	*30*	*0*.*04*	*−0*.*68*	*0*.*76*	*0*.*911*	*−*	*−*	*−*	*−*
			*PIP*	*1*	*131*	*0*.*05*	*−0*.*29*	*0*.*39*	*0*.*775*	***−***	*−*	*−*	*−*
			*QUE*	*4*	*420*	*−0*.*30*	*−0*.*65*	*0*.*04*	*0*.*085*	*0.143*	*44.7*	*−*	*−*
			*RIS*	***2***	***282***	***−0.48***	***−0.71***	***−0.24***	***<0.001***	*0.751*	*0.0*	*−*	*−*
			***ZIP***	***2***	***199***	***−0.44***	***−0.72***	***−0.15***	***0.003***	*0.536*	*0.0*	*−*	*−*
Safety	At least one AE		**ARI**	**6**	**2122**	**1.24**	**1.16**	**1.32**	**<0.001**	0.598	0.0		**8 (6–11)**
			**BRE**	**6**	**2244**	**1.24**	**1.11**	**1.39**	**<0.001**	0.123	42.3		**10 (6–22)**
			**CAR**	**1**	**812**	**1.25**	**1.12**	**1.40**	**<0.001**	−	−		**7 (5–13)**
			**ILO**	**1**	**26**	**2.45**	**1.27**	**4.74**	**0.008**	−	−		**2 (2–3)**
			OLA	**1**	126	1.22	0.80	1.86	0.352	−	−		NA
			PIP	1	163	1.11	0.99	1.24	0.078	−	−		NA
			**QUE**	**3**	**1242**	**1.25**	**1.14**	**1.37**	**<0.001**	0.501	0.0		**7 (5–13)**
			RIS	2	365	0.94	0.77	1.15	0.555	0.181	44.1		NA
			THI	1	78	1.40	0.54	3.67	0.489	−	−		NA
			ZIP	2	200	1.57	0.80	3.07	0.187	**0.023**	**80.7**		NA
	Any extrapyramidal AEs	Any EPS	BRE	1	394	1.65	0.87	3.14	0.124	−	−		NA
			QUE	2	936	1.19	0.63	2.24	0.597	0.348	0.0		NA
		Akathisia	**ARI**	**7**	**2610**	**3.57**	**2.06**	**6.18**	**<0.001**	**<0.001**	**76.5**		**7 (4–15)**
			**BRE**	**6**	**2244**	**2.97**	**1.94**	**4.55**	**<0.001**	0.314	15.6		**17 (10–36)**
			**CAR**	**1**	**812**	**6.41**	**2.83**	**14.5**	**<0.001**	−	−		**9 (7–12)**
			OLA	1	126	0.97	0.20	4.62	0.968	−	−		NA
			QUE	2	751	1.92	0.57	6.44	0.290	0.615	0.0		NA
			RIS	1	268	2.87	0.12	69.8	0.517	−	−		NA
			ZIP	2	200	2.34	0.91	6.04	0.078	0.533	0.0		NA
		Restlessness	**ARI**	**3**	**1085**	**4.48**	**2.42**	**8.30**	**<0.001**	0.344	6.3		**12 (6–30)**
			**BRE**	**5**	**1872**	**4.13**	**1.24**	**13.77**	**0.021**	0.079	52.2		**31 (8–392)**
			**CAR**	**1**	**812**	**3.06**	**1.40**	**6.71**	**0.005**	−	−		**19 (12–41)**
			QUE	2^d^	751	1.25	0.24	6.35	0.791	−	−		NA
		Tremor	**ARI**	**4**	**1550**	**2.19**	**1.06**	**4.52**	**0.035**	0.157	42.3		**28 (10–566)**
			BRE	1	449	1.65	0.66	4.11	0.284	−	−		NA
			**CAR**	**1**	**812**	**4.14**	**1.48**	**11.5**	**0.007**	−	−		**22 (14–45)**
			ILO	1	26	5.00	0.26	95.0	0.284	−	−		NA
			PIP	1	163	1.45	0.42	4.93	0.556	−	−		NA
			RIS	1	268	0.96	0.06	15.13	0.975	−	−		NA
			ZIP	1	61	3.41	0.45	25.9	0.235	−	−		NA
	Arousal-related AEs	Insomnia	ARI	7	2470	1.61	0.82	3.18	0.165	**0.025**	**58.6**		NA
			BRE	6	2244	1.79	0.92	3.48	0.087	0.189	33.0		NA
			**CAR**	**1**	**812**	**1.95**	**1.15**	**3.30**	**0.013**	−	−		**18 (11–57)**
			OLA	1	126	0.48	0.09	2.55	0.392	−	−		NA
			QUE	4	832	0.32	0.03	2.93	0.311	**0.034**	**70.4**		NA
			RIS	3	388	0.73	0.16	3.31	0.688	0.122	52.5		NA
			ZIP	2	200	2.14	0.32	14.2	0.430	0.085	66.3		NA
		Sedation	BRE	4^d^	1625	1.34	0.15	12.0	0.793	0.250	24.6		NA
			ILO	1	26	9.00	0.53	152	0.128	−	−		**4 (2–23)**
			**QUE**	**4**	**1356**	**3.68**	**2.25**	**6.01**	**<0.001**	0.568	0.0		**13 (7–27)**
		Somnolence	ARI	5	2071	1.39	0.84	2.29	0.197	0.259	24.4		NA
			**BRE**	**4**	**1625**	**4.25**	**1.56**	**11.6**	**0.005**	0.176	39.3		**26 (8–147)**
			**CAR**	**1**	**812**	**1.91**	**1.06**	**3.45**	**0.032**	−	−		**23 (13–112)**
			ILO	1	26	1.00	0.16	6.07	1.000	−	−		NA
			OLA	1	126	1.94	0.37	10.2	0.435	−	−		NA
			**QUE**	**4**	**1265**	**7.95**	**4.63**	**13.7**	**<0.001**	0.653	0.0		**6 (4–12)**
			RIS	2	291	2.76	0.77	9.91	0.120	0.667	0.0		NA
			**ZIP**	**2**	**200**	**2.66**	**1.39**	**5.10**	**0.003**	0.641	0.0		**6 (3–23)**
	Metabolic and endocrine AEs	⩾7% weight gain	**ARI**	**5**	**2083**	**5.96**	**3.14**	**11.3**	**<0.001**	0.963	0.0		**19 (10–44)**
			**BRE**	**4**	**1618**	**2.31**	**1.24**	**4.31**	**0.009**	0.848	0.0		**45 (18–247)**
			CAR	1	808	1.26	0.45	3.50	0.655	−	−		NA
			**QUE**	**4**	**1272**	**2.57**	**1.25**	**5.29**	**0.011**	0.842	0.0		**34 (13–215)**
			RIS	1	97	2.62	0.13	52.95	0.531				NA
		*Body weight change*	***ARI***	***5***	***1904***	***0.67***	***0.54***	***0.80***	***<0.001***	*0.118*	*45.7*	*−*	*−*
			***BRE***	***1***	***373***	***0.50***	***0.29***	***0.71***	***<0.001***	*−*	*−*	*−*	*−*
			***CAR***	***1***	***808***	***0.44***	***0.29***	***0.58***	***<0.001***	*−*	*−*	*−*	*−*
			***OLA***	***1***	***121***	***0.94***	***0.57***	***1.32***	***<0.001***	*−*	*−*	*−*	*−*
			***PIP***	***1***	***131***	***1.57***	***1.18***	***1.96***	***<0.001***	*−*	*−*	*−*	*−*
			***QUE***	***3***	***1050***	***0.37***	***0.24***	***0.49***	***<0.001***	*0.797*	*0.0*	*−*	*−*
			***RIS***	***2***	***120***	***0.79***	***0.40***	***1.18***	***<0.001***	*0.421*	*0.0*	*−*	*−*
			*ZIP*	*1*	*139*	*0*.*26*	*−0*.*07*	*0*.*60*	*0*.*124*	*−*	*−*	*−*	*−*
		*Glucose change*	*CAR*	*1*	*790*	*0*.*01*	*−0*.*14*	*0*.*15*	*0*.*930*	*−*	*−*	−	−
			*QUE*	*2*	*936*	*−0*.*03*	*−0*.*16*	*0*.*11*	*0*.*690*	*0.338*	*0.0*	−	−
			*ZIP*	*1*	*139*	*−0*.*06*	*−0*.*39*	*0*.*28*	*0*.*737*	*−*	*−*	−	−
		*HDL cholesterol change*	*CAR*	*1*	*790*	*−0*.*14*	*−0*.*28*	*0*.*01*	*0*.*073*	*−*	*−*	−	−
			*OLA*	*1*	*122*	*0*.*11*	*−0*.*25*	*0*.*46*	*0*.*553*	*−*	*−*	−	−
			***QUE***	***2***	***936***	***0.16***	***0.02***	***0.30***	***0.022***	*0.499*	*0.0*	−	−
			*ZIP*	*1*	*139*	*0*.*10*	*−0*.*23*	*0*.*43*	*0*.*548*	*−*	*−*	−	−
		Increased appetite	ARI	1	586	1.33	0.36	4.96	0.671	−	−		NA
			**BRE**	**2**	**775**	**3.81**	**1.43**	**10.2**	**0.008**	0.571	0.0		**28 (9–185)**
			CAR	1	812	2.31	0.80	6.73	0.124	−	−		NA
			ILO	1	26	3.00	0.13	67.5	0.489	−	−		NA
			QUE	4	832	1.25	0.58	2.68	0.564	0.246	27.6		NA
			RIS	2	120	2.00	0.09	44.66	0.661	0.084	66.5		NA
		*LDL cholesterol change*	*CAR*	*1*	*789*	*0*.*03*	*−0*.*11*	*0*.*18*	*0*.*660*	*−*	*−*	*−*	*−*
			*OLA*	*1*	*122*	*0*.*34*	*−0*.*02*	*0*.*70*	*0*.*063*	*−*	*−*	*−*	*−*
			*QUE*	*2*	*936*	*0*.*11*	*−0*.*03*	*0*.*24*	*0*.*125*	*0.365*	*0.0*	*−*	*−*
			*ZIP*	*1*	*139*	*0*.*00*	*−0*.*33*	*0*.*34*	*0*.*981*	*−*	*−*	*−*	*−*
		*Prolactin change*	***ARI***	***1***	***412***	***−0.56***	***−0.76***	***−0.37***	***<0.001***	*−*	*−*	*−*	*−*
			***CAR***	***1***	***790***	***0.35***	***0.20***	***0.50***	***<0.001***	*−*	*−*	*−*	*−*
			*QUE*	*2*	*936*	*−0*.*05*	*−0*.*19*	*0*.*08*	*0*.*456*	*0.761*	*0.0*	*−*	*−*
			*RIS*	*1*	*15*	*0*.*85*	*−0*.*21*	*1*.*91*	*0*.*114*	*−*	*−*	*−*	*−*
			*ZIP*	*1*	*139*	*−0*.*11*	*−0*.*44*	*0*.*22*	*0*.*512*	*−*	*−*	*−*	*−*
		Sexual dysfunction	QUE	1	445	1.99	0.22	17.7	0.536	−	−		NA
			ZIP	1	139	1.34	0.45	4.02	0.601	−	−		NA
		*Total cholesterol change*	*CAR*	*1*	*790*	*0*.*07*	*−0*.*08*	*0*.*22*	*0*.*343*	*−*	*−*	*−*	*−*
			***OLA***	***1***	***122***	***0.43***	***0.07***	***0.79***	***0.020***	*−*	*−*	*−*	*−*
			***QUE***	***2***	***936***	***0.20***	***0.06***	***0.34***	***0.004***	*0.869*	*0.0*	*−*	*−*
			*ZIP*	*1*	*139*	*−0*.*19*	*−0*.*52*	*0*.*14*	*0*.*260*	*−*	*−*	*−*	*−*
		*Triglycerides change*	*CAR*	*1*	*790*	*0*.*05*	*−0*.*10*	*0*.*19*	*0*.*543*	*−*	*−*	*−*	*−*
			*OLA*	*1*	*126*	*0*.*16*	*−0*.*19*	*0*.*51*	*0*.*359*	*−*	*−*	*−*	*−*
			***QUE***	***2***	***936***	***0.30***	***0.17***	***0.44***	***<0.001***	*0.593*	*0.0*	*−*	*−*
			*ZIP*	*1*	*139*	*0*.*05*	*−0*.*28*	*0*.*38*	*0*.*762*	*−*	*−*	*−*	*−*
		Weight increased	**ARI**	**3**	**1171**	**3.99**	**1.36**	**11.7**	**0.012**	0.211	35.8		**17 (5–135)**
			**BRE**	**5**	**1841**	**4.22**	**2.41**	**7.38**	**<0.001**	0.596	0.0		**20 (10–44)**
			**OLA**	**1**	**126**	**3.55**	**1.04**	**12.1**	**0.043**	*−*	*−*		**9 (5–60)**
			**QUE**	**2**	**503**	**3.87**	**1.39**	**10.7**	**0.010**	0.626	0.0		**16 (5–114)**
			RIS	2	365	2.09	0.56	7.78	0.272	0.481	0.0		NA
			ZIP	1	139	0.24	0.03	2.09	0.196	−	−		NA
	Suicide related outcomes	Suicidal attempt	ARI	1	586	1.00	0.09	10.9	0.998	−	−		NA
			BRE	1	379	NA^e^	NA	NA	NA	−	−		NA
			QUE	1	23	3.25	0.15	72.4	0.457	−	−		NA
			RIS	1	268	0.32	0.01	7.76	0.483	−	−		NA
		Suicidal ideation	ARI	2	706	0.72	0.05	10.3	0.807	0.226	31.7		NA
			BRE	4^f^	1625	0.58	0.32	1.05	0.073	0.864	0.0		NA
			CAR	1	808	1.10	0.65	1.85	0.724	−	−		NA
			QUE	2	751	0.70	0.21	2.27	0.549	0.613	0.0		NA
			ZIP	1	139	2.87	0.12	69.4	0.516	−	−		NA

AE, adverse event; AMI, amisulpride; AP, antipsychotics; ARI, aripiprazole; BRE, brexpiprazole; CAR, cariprazine; CI, confidence interval; EPS, extrapyramidal symptoms; ILO, iloperidone; LUR, lurasidone; *N*, the number of studies; *n*, the number of patients; NA, not applicable; NNH, number-needed-to-harm; NNT, number-needed-to-treat; OFC, olanzapine/fluoxetine combination; OLA, olanzapine; PER, perphenazine; PIP, pipamperone; QUE, quetiapine; RIS, risperidone; RR, risk ratio; SAE, serious adverse event; SMD, standardized mean difference; SUL, sulpiride; THI, thioridazine; ZIP, ziprasidone.

*Notes:* Significant (*p* < 0.05) results are in bold. Continuous data (SMD) are in italics.

RR values >1 indicate superiority of antipsychotics compared to placebo for positive outcomes, remission, while RR values >1 indicate inferiority of antipsychotics compared to placebo for negative outcomes (such as all cause discontinuation and discontinuation due to lack of efficacy).

SMDs <0 indicate superiority of antipsychotics compared to placebo in depressive symptom scale score. SMDs >0 indicate that antipsychotics had higher laboratory values than placebo.

a NNTs for remission and NNHs for other negative outcomes were calculated.

b NNTs for negative outcomes indicate that the placebo was more harmful than the antipsychotics.

c Includes a study (Liebowitz, 2010) with an incidence risk of 0% in both antipsychotic and placebo arms.

d Includes a study (Hobart, 2018) with an incidence risk of 0% in both antipsychotic and placebo arms.

e RR was not calculable as an incidence risk was 0% in both antipsychotic and placebo arms (Thase, 2015a).

f Includes a study (Thase, 2015b) with an incidence risk of 0% in both antipsychotic and placebo arms.

### Subgroup and meta-regression analyses

Table 2 summarizes the results of subgroup analyses for the co-primary outcomes. In monotherapy, significantly greater treatment response *v.* placebo was observed in subgroups of trials where antipsychotics were used at doses below DDD. Conversely, antipsychotics did not significantly separate from placebo regarding treatment response in a subgroup of trials where antipsychotics were used at doses higher than DDD. Such a relationship was not observed in adjunctive therapy. Online Supplementary Table S5 summarizes the results of meta-regression analyses for the co-primary outcomes. Significant correlations were found regarding mean age, % Caucasian, and number of depressive episodes in past year for treatment response in monotherapy; mean age and the number of lifetime depressive episode for treatment response in adjunctive therapy; as well as for sample size and DDD ratio for intolerability-related discontinuation in adjunctive therapy (online Supplementary Figs S2 and S3).

**Table 2.** Results of subgroup analysis for primary outcomes

Administration	Outcomes	Variables	Subgroup	*N*	*n*	RR	95% CI		*p*-value	Heterogeneity		NNT^a^ (95% CI)	NNH ^a^ (95% CI)
							Lower limit	Upper limit		*p*-value	*I*^2^		
Monotherapy	Treatment response	Overall		**10**	**2733**	**1.54**	**1.33**	**1.78**	**<0.001**	**0.014**	**56.5**	**5 (4–9)**	
		Sponsorship	**Academia**	**4**	**477**	**1.75**	**1.34**	**2.29**	**<0.001**	0.263	24.7	**5 (3–10)**	
			**Industry**	**5**	**2087**	**1.48**	**1.23**	**1.78**	**<0.001**	**0.008**	**70.7**	**6 (4–12)**	
			**NR**	**1**	**169**	**1.50**	**1.03**	**2.17**	**0.032**	−	−	**7 (4–57)**	
		Location	**Europe**	**3**	**480**	**1.82**	**1.48**	**2.24**	**<0.001**	0.409	0.0	**4 (3–7)**	
			Multi regions	2	642	1.57	0.89	2.76	0.116	**0.001**	**91.7**	NA	
			**North America**	**5**	**1611**	**1.41**	**1.24**	**1.60**	**<0.001**	0.654	0.0	**7 (5–12)**	
		Publication year	**1999 or earlier**	**3**	**480**	**1.82**	**1.48**	**2.24**	**<0.001**	0.409	0.0	**4 (3–7)**	
			**2000–2009**	**2**	**1146**	**1.52**	**1.28**	**1.80**	**<0.001**	0.974	0.0	**6 (4–11)**	
			**2010 or later**	**5**	**1107**	**1.41**	**1.08**	**1.83**	**0.011**	**0.007**	**71.7**	**7 (3–31)**	
		Antipsychotic drug category	**FGA**	**1**	**169**	**1.50**	**1.03**	**2.17**	**0.032**	−	−	**7 (4–57)**	
			**SGA**	**9**	**2564**	**1.55**	**1.32**	**1.81**	**<0.001**	**0.008**	**61.3**	**5 (4–9)**	
		Dosing	**Fixed**	**4**	**1457**	**1.66**	**1.43**	**1.93**	**<0.001**	0.339	10.7	**5 (4–7)**	
			**Flexible**	**6**	**1276**	**1.43**	**1.14**	**1.78**	**0.002**	**0.014**	**64.9**	**6 (4–18)**	
		AP dose^b^	**DDD ratio ⩽0.25**	**4**	**836**	**1.6749**	**1.3824**	**2.0293**	**<0.001**	0.273	23.0	**5 (3–9)**	
			**0.25 < DDD ratio** ⩽**0.5**	**5**	**1586**	**1.51**	**1.23**	**1.84**	**<0.001**	**0.005**	**72.7**	**5 (4–12)**	
			**0.5 < DDD ratio ⩽0.75**	**2**	**653**	**1.50**	**1.25**	**1.81**	**<0.001**	0.881	0.0	**6 (4–13)**	
			0.75 < DDD ratio ⩽1.0	0									
			DDD ratio >1.0	2	166	1.27	0.81	1.99	0.299	0.374	0.0	NA	
		Study quality^c^	**ROB low**	**7**	**2462**	**1.53**	**1.30**	**1.78**	**<0.001**	**0.012**	**63.4**	**6 (4–9)**	
			**ROB high**	3	271	1.57	1.00	2.48	0.050	0.158	45.8	NA	
	Discontinuation due to adverse event	Overall		**12**	**3693**	**2.56**	**1.86**	**3.54**	**<0.001**	0.912	0.0		**21 (13–38)**
		Sponsorship	**Academia**	**4**	**541**	**2.63**	**1.01**	**6.80**	**0.047**	0.868	0.0		**27 (8–3238)**
			**Industry**	**7**	**2975**	**2.59**	**1.84**	**3.66**	**<0.001**	0.646	0.0		**19 (12–36)**
			NR	1	177	1.03	0.07	16.3	0.981	−	−		NA
		Location	Europe	3	535	2.97	0.95	9.32	0.062	0.700	0.0		NA
			**Multiple regions**	**3**	**1428**	**2.29**	**1.46**	**3.61**	**<0.001**	0.333	9.0		**19 (10–54)**
			**North America**	**6**	**1730**	**3.02**	**1.75**	**5.19**	**<0.001**	0.884	0.0		**19 (9–50)**
		Publication year	1999 or earlier	3	535	2.97	0.95	9.32	0.062	0.700	0.0		NA
			**2000–2009**	**2**	**1184**	**3.49**	**1.69**	**7.17**	**0.001**	0.373	0.0		**18 (7–62)**
			**2010 or later**	**7**	**1974**	**2.32**	**1.59**	**3.39**	**<0.001**	0.829	0.0		**21 (12–46)**
		Antipsychotic drug category	FGA	2	230	1.55	0.26	9.23	0.629	0.706	0.0		NA
			**SGA**	**10**	**3463**	**2.61**	**1.88**	**3.62**	**<0.001**	0.842	0.0		**20 (12–36)**
		Dosing	**Fixed**	**5**	**1595**	**3.41**	**1.87**	**6.25**	**<0.001**	0.907	0.0		**19 (9–53)**
			**Flexible**	**7**	**2098**	**2.29**	**1.56**	**3.35**	**<0.001**	0.790	0.0		**22 (13–51)**
		AP dose^b^	DDD ratio ⩽0.25	6	1185	1.88	0.86	4.15	0.116	0.659	0.0		NA
			**0.25 < DDD ratio** ⩽**0.5**	**6**	**2409**	**2.68**	**1.80**	**4.01**	**<0.001**	0.303	17.1		**18 (10–38)**
			**0.5 < DDD ratio ⩽0.75**	**2**	**672**	**3.08**	**1.41**	**6.75**	**0.005**	0.417	0.0		**21 (8–104)**
			0.75 < DDD ratio ⩽1.0	0									
			DDD ratio >1.0	2	183	1.65	0.38	7.11	0.500	0.894	0.0		NA
		Study quality^c^	**ROB low**	**7**	**2535**	**3.18**	**2.10**	**4.82**	**<0.001**	0.951	0.0		**18 (10–35)**
			**ROB high**	**5**	**1158**	**1.85**	**1.11**	**3.09**	**0.018**	0.883	0.0		**31 (13–231)**
Adjunctive therapy	Treatment response	Overall		**28**	**7366**	**1.35**	**1.26**	**1.45**	**<0.001**	0.440	1.6	**12 (9–16)**	
		Sponsorship	Academia	0	−	−	−	−	−	−	−	−	
			**Industry**	**28**	**7366**	**1.35**	**1.26**	**1.45**	**<0.001**	0.440	1.6	**12 (9–16)**	
		Location	**Asia**	**2**	**595**	**1.45**	**1.13**	**1.86**	**0.004**	0.814	0.0	**8 (5–28)**	
			Europe	2	114	0.99	0.78	1.25	0.900	0.459	0.0	NA	
			**Multiple regions**	**8**	**3568**	**1.30**	**1.17**	**1.43**	**<0.001**	0.835	0.0	**15 (10–25)**	
			**North America**	**15**	**3069**	**1.48**	**1.33**	**1.65**	**<0.001**	0.640	0.0	**9 (7–13)**	
			ROTW	1	20	2.00	0.88	4.54	0.098	−	−	**3 (2–125)**	
		Publication year	1999 or earlier	1	78	0.95	0.74	1.22	0.696	−	−	NA	
			**2000–2009**	**9**	**2003**	**1.50**	**1.32**	**1.70**	**<0.001**	0.393	5.0	**8 (6–12)**	
			**2010 or later**	**18**	**5285**	**1.34**	**1.23**	**1.46**	**<0.001**	0.929	0.0	**13 (10–20)**	
		Antipsychotic drug category	FGA	1	78	0.95	0.74	1.22	0.696			NA	
			**SGA**	**27**	**7288**	**1.39**	**1.29**	**1.49**	**<0.001**	0.815	0.0	**11 (9–15)**	
		Dosing	**Fixed**	**10**	**2573**	**1.20**	**1.08**	**1.34**	**0.001**	0.418	2.3	**18 (11–44)**	
			**Flexible**	**17**	**4207**	**1.48**	**1.35**	**1.63**	**<0.001**	0.901	0.0	**10 (8–13)**	
			**Fixed/flexible**^c^	**1**	**586**	**1.44**	**1.12**	**1.86**	**0.005**	−	−	**8 (5–23)**	
		AP dose^b^	**DDD ratio ⩽0.25**	**6**	**990**	**1.47**	**1.21**	**1.78**	**<0.001**	0.748	0.0	**10 (6–21)**	
			**0.25 < DDD ratio** ⩽**0.5**	**12**	**2746**	**1.28**	**1.14**	**1.43**	**<0.001**	0.248	19.9	**12 (8–24)**	
			**0.5 < DDD ratio ⩽0.75**	**9**	**3388**	**1.36**	**1.23**	**1.49**	**<0.001**	0.398	4.4	**10 (8–16)**	
			**0.75 < DDD ratio ⩽1.0**	**3**	**805**	**1.45**	**1.14**	**1.84**	**0.003**	0.770	0.0	**12 (6–37)**	
			**DDD ratio >1.0**	**3**	**219**	**1.86**	**1.13**	**3.06**	**0.015**	0.747	0.0	**7 (3–48)**	
		Study quality^c^	**ROB low**	**15**	**6008**	**1.36**	**1.26**	**1.47**	**<0.001**	0.541	0.0	**12 (9–16)**	
			**ROB high**	**13**	**1358**	**1.37**	**1.14**	**1.64**	**0.001**	0.279	16.4	**12 (7–30)**	
		Concealment	**Double blinded**	**26**	**7286**	**1.35**	**1.26**	**1.45**	**<0.001**	0.417	3.2	**12 (10–16)**	
			**Single blinded/OL**	**2**	**80**	**2.11**	**1.04**	**4.30**	**0.039**	0.790	0.0	**5 (2–127)**	
	Discontinuation due to adverse event	Overall		**27**	**7778**	**2.39**	**1.69**	**3.38**	**<0.001**	0.100	27.3		**37 (22–73)**
		Sponsorship	Academia	1	181	0.99	0.21	4.77	0.989	−	−		NA
			**Industry**	**26**	**7597**	**2.47**	**1.73**	**3.52**	**<0.001**	0.100	27.7		**35 (21–70)**
		Location	Asia	2	627	2.11	0.73	6.06	0.167	0.291	10.5		NA
			Europe	2	243	0.39	0.10	1.46	0.161	0.650	0.0		NA
			**International**	**8**	**3657**	**3.00**	**1.93**	**4.66**	**<0.001**	0.900	0.0		**35 (19–75)**
			**North America**	**15**	**3251**	**2.72**	**1.56**	**4.74**	**<0.001**	0.058	40.5		**27 (13–81)**
		Publication year	1999 or earlier	1	78	0.26	0.03	2.25	0.223	−	−		NA
			**2000–2009**	**8**	**2071**	**2.29**	**1.27**	**4.13**	**0.006**	0.113	39.9		**28 (12–132)**
			**2010 or later**	**18**	**5629**	**2.69**	**1.79**	**4.04**	**<0.001**	0.332	10.4		**38 (22–81)**
		Antipsychotic drug category	FGA	2	243	0.39	0.10	1.46	0.161	0.650	0.0		NA
			**SGA**	**25**	**7535**	**2.60**	**1.88**	**3.58**	**<0.001**	0.267	14.0		**35 (22–62)**
		Dosing	**Fixed**	**10**	**2733**	**2.61**	**1.14**	**6.01**	**0.024**	**0.032**	**52.4**		**33 (11–382)**
			**Flexible**	**16**	**4459**	**2.24**	**1.54**	**3.26**	**<0.001**	0.316	12.0		**38 (21–88)**
			Fixed/flexible^d^	1	586	3.74	0.86	16.2	0.078	−	−		NA
		AP dose^b^	**DDD ratio ⩽0.25**	3	598	1.40	0.45	4.28	0.560	0.195	38.9		NA
			**0.25 < DDD ratio** ⩽**0.5**	**9**	**2084**	**2.01**	**1.06**	**3.81**	**0.032**	0.054	47.6		**32 (12–521)**
			**0.5 < DDD ratio ⩽0.75**	**8**	**3061**	**4.49**	**2.86**	**7.05**	**<0.001**	0.769	0.0		**20 (12–38)**
			0.75 < DDD ratio ⩽1.0	2	809	2.02	0.82	4.96	0.125	0.454	0.0		NA
			**DDD ratio >1.0**	**3**	**220**	**17.6**	**3.51**	**88.7**	**<0.001**	0.993	0.0		**4 (3–11)**
		Study quality^c^	**ROB low**	**17**	**6536**	**2.79**	**1.99**	**3.90**	**<0.001**	0.441	1.0		**36 (22–64)**
			ROB high	10	1242	1.80	0.85	3.81	0.123	0.071	44.7		NA
		Concealment	**Double blinded**	**26**	**7717**	**2.32**	**1.64**	**3.27**	**<0.001**	0.116	26.0		**38 (22–78)**
			**Single blinded/OL**	**1**	**61**	**16.5**	**1.04**	**262**	**0.047**	−	−		**3 (2–5)**

AP, antipsychotic drug; CI, confidence interval; DDD, defined daily dose; FGA, first-generation antipsychotic drug; *N*, the number of studies; *n*, the number of patients; NA, not applicable; NNH, number-needed-to-harm; NNT, number-needed-to-treat; NR, not reported; OL, open label; ROB, risk of bias; ROTW, rest of the world; RR, risk ratio; SGA, second-generation antipsychotic drug; UK, United Kingdom; US, United States of America.

*Notes*: Significant (*p* < 0.05) results are in bold.

a NNTs for treatment response and NNHs for discontinuation due to adverse event were calculated.

b Where a study involved more than two appropriate dose groups of an antipsychotic drug, the different dose arms were treated separately.

c Risk of bias was evaluated in accordance with the Cochrane Handbook for Systematic Reviews of interventions. High quality RCTs were defined as the number of low risk ratings more than five domains (=ROB low), whereas low-quality RCTs less than four domains (ROB high).

d Data on aripiprazole 3 mg/day (fixed dose) and aripiprazole 3–15 mg/day (flexible dose) group reported in Kamijima (2013) were combined.

### Publication bias

In 8 of 13 comparisons with ⩾3 studies, the funnel-plot was asymmetrical. Trim-and-fill did not alter the results (online Supplementary Fig. S4).

## Discussion

To the best of our knowledge, this is the largest meta-analysis examining the efficacy and safety/tolerability of antipsychotic treatment for depression both as monotherapy including data from 13 RCTs including six different antipsychotics and 4375 participants, and as adjunctive therapy including 32 RCTs including 11 different antipsychotics and 8349 participants. These results represent the first meta-analysis of antipsychotic monotherapy for MDD and a meaningful increase of two-fold (Spielmans et al., 2013) to more than two-fold (Nelson & Papakostas, 2009) in terms of number of studies (32 *v.* 14 and 16) and more than two-fold in terms of number of participants (8349 *v.* 3480 and 3549) compared to the two prior largest meta-analyses of adjunctive antipsychotic therapy for MDD (Nelson & Papakostas, 2009; Spielmans et al., 2013). In monotherapy, the pooled effect sizes for antipsychotics were RR = 1.54 (95% CI 1.33–1.78, *p* < 0.001, NNT = 5) for treatment response and RR = 2.56 (95% CI 1.86–3.54, *p* < 0.001, NNH = 21) for intolerability-related discontinuation, indicating a significant benefit of using antipsychotics regarding efficacy, but also a significantly higher risk for adverse events. Therefore, these results question the need for antipsychotic monotherapy in the treatment of MDD, given the higher risk compared to benefit and availability of both safe and effective antidepressants (Cipriani et al., 2018). Nevertheless, individually, sulpiride showed a favorable risk/benefit balance [efficacy RR = 1.50 (95% CI 1.03–2.17, *p* = 0.032), tolerability RR = 1.03 (95% CI 0.07–16.3, *p* = 0.981)].

In adjunctive therapy, the effect sizes of antipsychotics overall were RR = 1.35 (95% CI 1.26–1.45, *p* < 0.001, NNT = 12) for treatment response and RR = 2.39 (95% CI 1.69–3.38, *p* < 0.001, NNH = 37) for intolerability-related discontinuation, again indicating larger risk than benefit. Even looking at individual antipsychotics, only risperidone showed a favorable risk/benefit balance [efficacy RR = 1.59 (95% CI 1.19–2.14, *p* = 0.002), tolerability RR = 1.15 (95% CI 0.29–4.53, *p* = 0.840)].

Sulpiride is a selective antagonist at dopamine D_2_, D_3_, and 5-HT_1A_ receptors (Caley & Weber, 1995; Hall, Sallemark, & Jerning, 1986). At doses of 600–1600 mg/day, sulpiride shows mild sedating and antipsychotic activity (Caley & Weber, 1995), whereas at low doses of around 300 mg/day, its prominent feature is antagonism of presynaptic inhibitory dopamine and serotonin receptors, accounting for some antidepressant activity and a monoaminergic stimulating effect (Pani & Gessa, 2002).

In adjunctive therapy, six out of nine antipsychotics were associated with significantly higher treatment response than placebo. Preclinical reports indicate that SGAs modulate the monoaminergic neurotransmitter systems thought to be involved in antidepressant mechanisms; i.e. presynaptic serotonin and/or dopamine blockade, leading to the increase of postsynaptic dopamine release (Blier & Szabo, 2005; Hertel, Nomikos, & Svensson, 1997; Tremblay & Blier, 2006). Conversely, intolerability-related discontinuation was prominent with antipsychotics; presumably due to antipsychotics-induced adverse events, such as akathisia, EPS, sedation, weight gain, etc.

Notably, in our sensitivity analysis that examined the antipsychotic dose for antidepressant effect, the high-dose range did not have treatment effect when antipsychotics were used as monotherapy, whereas in low- or middle-dose range they did. Presumably, this is because at higher doses, antipsychotics potently inhibited dopamine D_2_ receptors, thereby dampening the dopaminergic reward system and increasing their sedative effects; whereas at low doses, the relative affinity for serotonin 5-HT_2_ receptors is more potent than for dopamine D_2_ receptors (Schotte et al., 1996). When used adjunctively, this dose relationship was not prominent, indicating this mechanism of action may be more complex when antipsychotics are combined with antidepressants. Nevertheless, it is notable that the utilized mean dosages of risperidone were low (0.8–2.0 mg/day). Additionally, at higher antipsychotic doses, the intolerability-related discontinuations were also higher. Thus, depending on the specific SGA and its doses, as well as monotherapy/adjunctive use, antidepressant effects of antipsychotics can differ significantly, emphasizing the need for a better understanding of the complex role for antipsychotics in the treatment of MDD.

### Limitations

There are several limitations to this meta-analysis. First, the number of RCTs for individual antipsychotics was small. This was especially noticeable for monotherapy. There were only two studies of amisulpride and one study of sulpiride; therefore, caution is needed in interpreting these results, and further clinical research would certainly be necessary. In addition, of the 13 monotherapy RCTs included in the meta-analysis, seven were quetiapine studies, so the meta-analysis results of monotherapy were heavily influenced by the quetiapine studies. There were also insufficient numbers of RCTs in some subgroups, including studies sponsored by academia, information with DDD ratio, etc. The trial duration for most of the RCTs was 6–8 weeks, so it is possible that there was insufficient variation to assess the effect of trial duration. Furthermore, the outpatient studies were in the majority, and there was a relatively large number of studies that did not report the in-/outpatient status of the included patients. Given this, as well as the fact that there were very few inpatient studies, it was difficult to examine the impact of treatment setting on antipsychotic drug efficacy and/or adverse effect. Second, we selected treatment response and intolerability-related discontinuation as the co-primary outcomes to evaluate the risk-benefit balance of antipsychotics. The results, however, can vary significantly depending on which outcomes are chosen. It should also be noted that criteria for treatment response are not always uniform between trials. Third, when evaluating the effect of adjunctive antipsychotic therapy, we regarded antidepressants as a group without distinguishing the specific antidepressant. It is possible that the therapeutic effect may vary depending on the combination of the specific antipsychotic and the specific antidepressant, but data were insufficient to examine this possibility.

## Conclusions

In summary, results of this to our knowledge currently largest meta-analysis of antipsychotic treatment for MDD suggest that antipsychotics were efficacious for MDD both as monotherapy and adjunctively, but that caution is needed, as the risks for intolerability-related discontinuations were higher than with placebo and generally higher than the effect sizes for efficacy. Nevertheless, the risk/benefit balance of antipsychotic varied by specific antipsychotic and their dose, with a favorable risk/benefit ratio in monotherapy for sulpiride and adjunctively for low-dose risperidone. These results should inform clinical use regarding the role of antipsychotics in the management of MDD.

## Supplementary material

For supplementary material accompanying this paper visit https://doi.org/10.1017/S0033291722000745. click here to view supplementary material
