# Supplementary material for: Efficacy and safety/tolerability of antipsychotics in the treatment of adult patients with major depressive disorder: a systematic review and meta-analysis
Source: Psychol Med. 2022 May 5;53(9):4064–82. doi: 10.1017/S0033291722000745 (PMC10317805; doi:10.1017/S0033291722000745)
Supplement: Supplementary file 1 [file S0033291722000745sup001.docx]

**Supplementary materials to Kishimoto et al.
Efficacy and safety/tolerability of antipsychotics in the treatment of adult patients with major depressive disorder: A systematic review and meta-analysis.**

***Psychological Medicine***

[**Table S1 Risk of bias summary** 2](#_Toc94946986)

[(“low”: low risk of bias; “?”: unclear risk of bias; “high“: high risk of bias) 3](#_Toc94946987)

[**Table S2 Characteristics of included studies** 4](#_Toc94946988)

[**Table S3 Key findings** 11](#_Toc94946989)

[**Table S4 Results of meta-analysis for secondary outcomes** 13](#_Toc94946990)

[**Table S5 Meta-regression analyses for co-primary outcomes** 22](#_Toc94946991)

[**Figure S1 Flow-diagram describing the search process** 24](#_Toc94946992)

[**Figure S2 Meta-regression analysis in monotherapy** 25](#_Toc94946993)

[**(A)** **Association between treatment response and mean age** 25](#_Toc94946994)

[**(B) Association between treatment response and % Caucasian** 26](#_Toc94946995)

[**(C) Association between treatment response and number of depressive episodes in past year** 27](#_Toc94946996)

[**Figure S3 Meta-regression analysis in adjunctive therapy** 28](#_Toc94946997)

[**(A) Association between treatment response and mean age** 28](#_Toc94946998)

[**(B) Association between treatment response and number of lifetime depressive episodes** 29](#_Toc94946999)

[**(C) Association between discontinuation due to adverse event and sample size** 30](#_Toc94947000)

[**(D) Association between discontinuation due to adverse event and DDD ratio** 31](#_Toc94947001)

[**Figure S4 Publication bias assessed by funnel plots, fail-safe estimates and Egger’s tests for the co-primary outcomes** 32](#_Toc94947002)

[**(A)** **Treatment response, monotherapy; all RCTs** 32](#_Toc94947003)

[**(B)** **Treatment response, monotherapy; RCTs of QUE** 33](#_Toc94947004)

[**(C)** **Discontinuation due to adverse event, monotherapy; all RCTs** 34](#_Toc94947005)

[**(D)** **Discontinuation due to adverse event, monotherapy; RCTs of QUE** 35](#_Toc94947006)

[**(E)** **Treatment response, adjunctive therapy; all RCTs** 36](#_Toc94947007)

[**(F)** **Treatment response, adjunctive therapy; RCTs of ARI** 37](#_Toc94947008)

[**(G)** **Treatment response, adjunctive therapy; RCTs of BRE** 38](#_Toc94947009)

[**(H)** **Treatment response, adjunctive therapy; RCTs of QUE** 39](#_Toc94947010)

[**(I)** **Discontinuation due to adverse event, adjunctive therapy; all RCTs** 40](#_Toc94947011)

[**(J)** **Discontinuation due to adverse event, adjunctive therapy; RCTs of ARI** 41](#_Toc94947012)

[**(K)** **Discontinuation due to adverse event, adjunctive therapy; RCTs of BRE** 42](#_Toc94947013)

[**(L)** **Discontinuation due to adverse event, adjunctive therapy; RCTs of QUE** 43](#_Toc94947014)

[**References** 44](#_Toc94947015)

**Table S1 Risk of bias summary**

|  | ***Random sequence generation*** | ***Allocation concealment*** | ***Blinding of participants and personnel*** | ***Blinding of outcome assessment*** | ***Incomplete outcome data addressed*** | ***Selective reporting*** | ***Other sources of bias*** |
| --- | --- | --- | --- | --- | --- | --- | --- |
| Monotherapy | | | | | | | |
| Bortnick 2011 | **?** | **?** | **low** | **low** | **low** | **low** | **low** |
| Boyer 1999 | **?** | **?** | **low** | **low** | **low** | **low** | **low** |
| Chaput 2008 | **?** | **low** | **low** | **low** | **high** | **low** | **?** |
| Cutler 2009 | **low** | **low** | **low** | **low** | **low** | **low** | **low** |
| Frolund 1974 | **?** | **?** | **low** | **low** | **low** | **?** | **low** |
| Katila 2013 | **low** | **?** | **low** | **low** | **low** | **low** | **low** |
| Kennedy 2014 | **?** | **?** | **low** | **low** | **high** | **?** | **low** |
| Lecrubier 1997 | **?** | **?** | **low** | **low** | **high** | **low** | **low** |
| Liebowitz 2010 | **?** | **?** | **low** | **low** | **high** | **low** | **low** |
| Papakostas 2012 | **?** | **?** | **low** | **low** | **low** | **low** | **high** |
| Rüther 1999 | **?** | **?** | **low** | **low** | **low** | **low** | **low** |
| Wang 2014 | **low** | **low** | **low** | **low** | **low** | **low** | **low** |
| Weisler 2009 | **low** | **low** | **low** | **low** | **low** | **low** | **low** |
| Adjunctive therapy | | | | | | | |
| Bauer 2009 | **?** | **?** | **low** | **low** | **low** | **low** | **low** |
| Berman 2007 | **low** | **low** | **low** | **low** | **low** | **low** | **low** |
| Berman 2009 | **?** | **?** | **low** | **low** | **low** | **low** | **low** |
| Dunner 2007 | **?** | **?** | **high** | **high** | **high** | **low** | **low** |
| Durgam 2016 | **low** | **low** | **low** | **low** | **low** | **low** | **low** |
| El-Khalili 2010 | **low** | **?** | **low** | **low** | **low** | **low** | **low** |
| Fava 2012 | **?** | **?** | **low** | **low** | **low** | **low** | **high** |
| Flint 2019 | **low** | **?** | **low** | **low** | **low** | **low** | **low** |
| Garakani 2008 | **?** | **?** | **low** | **low** | **?** | **low** | **low** |
| Hobart 2018a | **low** | **low** | **low** | **low** | **low** | **low** | **low** |
| Hobart 2018b | **low** | **low** | **low** | **low** | **low** | **low** | **low** |
| Ionesc 2016 | **?** | **?** | **low** | **low** | **high** | **low** | **high** |
| Kamijima 2013 | **?** | **?** | **low** | **low** | **low** | **low** | **low** |
| Kamijima 2018 | **?** | **?** | **low** | **low** | **low** | **low** | **low** |
| Keitner 2009 | **?** | **?** | **low** | **low** | **?** | **high** | **low** |
| Lenze 2015 | **low** | **?** | **low** | **low** | **low** | **low** | **low** |
| Li 2016 | **?** | **?** | **low** | **low** | **high** | **low** | **high** |
| Lin 2011 | **?** | **?** | **low** | **low** | **high** | **low** | **low** |
| Mahmoud 2007 | **low** | **low** | **low** | **low** | **low** | **low** | **low** |
| Marcus 2008 | **?** | **?** | **low** | **low** | **low** | **low** | **low** |
| McIntyre 2007 | **?** | **?** | **low** | **low** | **high** | **high** | **low** |
| Papakostas 2015 | **low** | **low** | **low** | **low** | **low** | **low** | **low** |
| Parker 2005 | **?** | **?** | **low** | **high** | **?** | **high** | **high** |
| Quante 2013 | **?** | **?** | **low** | **low** | **?** | **low** | **low** |
| Reeves 2008 | **?** | **?** | **low** | **low** | **?** | **low** | **low** |
| Sim 1978 | **?** | **?** | **low** | **low** | **?** | **low** | **low** |
| Stabl 1995 | **?** | **?** | **low** | **low** | **?** | **low** | **low** |
| Thase 2015a | **low** | **low** | **low** | **low** | **low** | **low** | **low** |
| Thase 2015b | **low** | **low** | **low** | **low** | **low** | **low** | **low** |
| Wade 2011 | **low** | **low** | **low** | **low** | **low** | **low** | **low** |
| NCT00797966 | **?** | **?** | **low** | **low** | **low** | **?** | **low** |
| NCT01052077 | **?** | **?** | **low** | **low** | **low** | **?** | **low** |

Risk of bias summary: review authors' judgements about each risk of bias item for each included study

(“low”: low risk of bias; “?”: unclear risk of bias; “high“: high risk of bias)

**Table S2 Characteristics of included studies**

| **Study/**  **Country/**  **Blinding status** | **Total no. of patients** | **Duration (weeks)** | **Patient characteristics**  **Diagnosis**  **Age** | | **Continuation**  **treatment(s)** | **Treatment arms (n)** | | **Mean age (years)** | **% Male** | **% White** | **Mean dose (mg/day)** |
| --- | --- | --- | --- | --- | --- | --- | --- | --- | --- | --- | --- |
| ***Monotherapy*** | | | | | | | | | | | |
| Bortnick 2011 (1)  US  DB | 310 | 8 | Diagnosis: MDD (DSM-IV),  Age: 18-65 years,  Severity: HAM-D-17 ≥ 22 and a HAM-D Item 1 (depressed mood) score ≥ 2 | | None | QUE (154)  PBO (156) | | 43.3  42.6 | 35.4  35.5 | 68.7  65.8 | 162.2 |
| Boyer 1999 (2)  France  DB | 323 | 12 | Diagnosis: Primary dysthymia (DSM-III-R)  Age: ≥ 18 years  Severity: MADRS < 21 | | None | AMI (104)  [AMIN (111)]  PBO (108) | | 48  48  48 | 29.8  21.6  24.1 | 99.0  98.2  99.1 | AMI 50 and AMIN 200 fixed dose |
| Chaput 2008 (3)  Canada  DB | 22 | 12 | Diagnosis: MDD (DSM-IV)  Age: 23-66 years  Severity: HAM-D-21 ≥ 18, CGI-S score ≥ 4, treatment refractory depression | | None | QUE (11)  PBO (11) | | 41.6  44.9 | 27.3  27.3 | NR | 147.7 |
| Cutler 2009 (4)  US  DB | 612 | 6 | Diagnosis: MDD (DSM-IV)  Age: 18-65 years,  Severity: HAM-D-17 ≥ 22 and HAM-D Item 1 score ≥ 2 | | None | QUE 150 (152)  QUE 300 (152)  [Duloxetine 60 (151)]  PBO (157) | | 40.9  41.6  40.2  42.3 | 36.7  49.0  37.6  35.5 | 75.5  74.8  75.9  69.1 | QUE: 150 and 300 fixed dose  Duloxetine: 60 fixed dose |
| Frolund 1974 (5)  Denmark  DB | 231 | 2 | Diagnosis: Depression (clinical diagnosis)  Age: 20-70 years  Severity: NR | | None | FLU (120)  PBO (111) | | NR | 15  20 | NR | 0.5 – 1.5 |
| Katila 2013 (6)  Multi  DB | 338 | 9 | Diagnosis: MDD (DSM-IV)  Age: ≥ 66 years  Severity: HAM-D-17 ≥ 22 and HAM-D Item 1 score ≥ 2 | | None | QUE (166)  PBO (172) | | 71.3  71.2 | 29.9  29.8 | 98.8  98.2 | 158.7 |
| Kennedy 2014 (7)  Canada  DB | 53 | 1 | Diagnosis: MDD (DSM-IV)  Age: 18-65 years  Severity: HAM-D-17 > 14 | | None | HAL (26)  PBO (27) | | NR | NR | NR | 0.25 |
| Lecrubier 1997 (8)  France  DB | 219 | 24 | Diagnosis: Primary dysthymia with major depression (DSM-III-R)  Age: NR  Severity: Mild or moderate severity or isolated chronic major depression in partial remission | | None | AMI (73)  [IMI (73)]  PBO (73) | | 41.8  44.0  42.9 | 43.8  52.1  39.7 | NR | AMI 50 and IMI 100 fixed dose |
| Liebowitz 2010 (9)  Multi  DB | 776 | 52 | Diagnosis: MDD (DSM-IV TR)  Age: 18-65 years  Severity: MADRS score ≤ 12 and CGI-S score ≤ 3 | | None | QUE (391)  PBO (385) | | 45.4  43.8 | 34.1  33.9 | NR | 177.1 |
| Papakostas 2012 (10)  US  DB | 120 | 12 | Diagnosis: MDD (DSM-IV)  Age: 18-65 years  Severity: QIDS-SR ≥ 10 | | None | ZIP-ZIP (29) ^a)^  PBO-ZIP (47) ^a)^  PBO-PBO (44) ^a)^ | | 41.5  44.1  44.6 | 55.2  61.7  50.0 | NR | Phase I 81.4  Phase II 113.8 |
| Rüther 1999 (11)  Germany and Austria  DB | 177 | 6 | Diagnosis: Depressive syndrome (ICD-10: F32.0, F32.1, F33.0, F33.1)  Age: 18-70 years  Severity: 27 ≥ HAM-D-21 ≥ 18 | | None | SUL (87)  PBO (90) | | 49.1  51.2 | 30.1  30.7 | NR | 181 |
| Wang 2014 (12)  Multi  DB | 471 | 8 | Diagnosis: MDD (DSM-IV)  Age: 18-65 years  Severity: HAM-D-17 ≥ 22 and HAM-D Item 1 ≥ 2 | | None | QUE (157)  [ESC (157)]  PBO (157) | | 40.1  40.3  39.7 | 28.6  24.3  32.7 | 55.8  52.6  54.9 | QUE: 139.8  ESC: 10.7 |
| Weisler 2009 (13)  US  DB | 723 | 6 | Diagnosis: MDD (DSM-IV)  Age: 18-65 years  Severity: HAM-D-17 ≥ 22 and HAM-D Item 1 ≥2 | | None | QUE 50 (182)  QUE 150 (178)  QUE 300 (179)  PBO (184) | | 40.6  41.5  40.7  40.3 | 46.6  38.1  41.5  36.5 | 73.6  73.8  69.9  76.4 | 50, 150 and 300 fixed dose |
| **- Number of studies:** 13 studies (13 reports)  **- Number of patients:** 4,375  [median=310 (range = 22−776)] **- Country:**  US (studies=4, n=1,765)  Multinational (studies=4, n=1,762) Canada (studies=2, n=75)  France (studies=2, n=542) Denmark (study=1, n=231) | | | | **- Blinding status:** DB (studies=13, n=4,375)  **- Mean duration:** 12.2 weeks (range **=** 1 − 52)  **- Antipsychotics:**  AMI (studies=2, n=542), FLU (study=1, n=231),  HAL (study=1, n=53), QUE (studies=7, n=3,252),  SUL (study=1, n=177), ZIP (study=1, n=120) | | | **- Age:** 45.2±7.6 (range=39.7 – 71.3) years  **- Gender** (Male 35.4%)  **- Race** (White 76.7%) | | | | |
| ***Adjunctive therapy*** | | | | | | | | | | | |
| Bauer 2009 (14)  Multi  DB | 493 | 6 | Diagnosis: MDD (DSM-IV)  Age: 18-65 years  Severity: HAM-D-17 ≥ 20, HAM-D Item 1 score ≥2, and history of an inadequate response during the current episode | | AD | QUE 150 (167)  QUE 300 (163)  PBO (163) | | 46.0  45.5  44.8 | 30.7  31.7  35.0 | 99.4  96.9  98.1 | 150 and 300 fixed dose |
| Berman 2007 (15)  US  DB | 362 | 6 | Diagnosis: MDD (DSM-IV),  Age: 18-65 years  Severity: HAM-D-17 ≥ 18, and inadequate response to AD treatment | | AD | ARI (182)  PBO (176) | | 46.5  44.2 | 38.5  35.8 | 87.4  92.6 | 11.8 |
| Berman 2009 (16)  US  DB | 349 | 6 | Diagnosis: MDD (DSM-IV)  Age: 18-65 years  Severity: Inadequate response to AD | | AD | ARI (177)  PBO (172) | | 45.1  45.6 | 22.0  32.0 | 87.6  86.6 | 10.7 |
| Dunner 2007 (17)  US  OL | 61 | 8 | Diagnosis: MDD (DSM-IV)  Age: 21-65 years  Severity: MADRS > 14, CGI-S ≥ 4, nonresponse to AD | | Sertraline | ZIP 80 (22)  ZIP 160 (19)  PBO (20) | | 43.1  42.6  46.3 | 45.5  52.6  45.0 | 90.9  94.7  80.0 | 80 and 160  Fixed dose |
| Durgam 2016 (18)  Multi  DB | 819 | 8 | Diagnosis: MDD (DSM-IV-TR) without psychotic features  Age: 18-65 years  Severity: Inadequate response to AD | | AD | CAR1-2 (274)  CAR 2-4.5 (276)  PBO (269) | | 45.5  45.1  46.4 | 31.5  26.4  28.6 | 85.7  88.6  86.5 | CAR 1 – 2: 1.4  CAR 2 – 4.5: 2.6 |
| El-Khalili 2010 (19)  US  DB | 446 | 6 | Diagnosis: MDD (DSM-IV)  Age: 18-65 years  Severity: HAM-D-17 ≥ 20 and HAM-D Item 1 ≥ 2 | | AD | QUE 150 (148)  QUE 300 (150)  PBO (148) | | 45.9  44.3  46.2 | 23.8  27.4  31.5 | 89.5  91.1  89.5 | 150 and 300 fixed dose |
| Fava 2012 (20)  US  DB | 225 | 4.3 | Diagnosis: MDD (DSM, SAFER)  Age: 18–65 years  Severity: QIDS-SR > 15, HAM-D-17 ≥ 18, inadequate response to AD | | AD | ARI-ARI (56) ^b)^  PBO-ARI (84) ^b)^  PBO-PBO (85) ^b)^ | | 45.1 | ARI  33.93  PBO  36.09 | ARI  83.93  PBO  79.88 | 2 or 5 |
| Flint 2019 (21)  US and Canada  DB | 126 | 36 | Diagnosis: MDD (DSM-IV-TR) with at least one associated delusion  Age: 18–85 years  Severity: Participants who met full-remission or near-remission criteria ^c)^ following treatment with sertraline plus olanzapine and who had a MMSE score ≥24 were eligible. | | Sertraline | OLA (64)  PBO (62) | | 55.0  55.7 | 42.2  33.9 | 84.4  79.0 | 5 – 20 |
| Garakani 2008 (22)  US  DB | 114 | 8 | Diagnosis: MDD (DSM-IV)  Age: 18-65 years  Severity: MADRS > 15 | | Fluoxetine | QUE (57)  PBO (57) | | NR | NR | NR | 47.3 |
| Hobart 2018a (23)  Multi  DB | 394 | 6 | Diagnosis: MDD (DSM-IV-TR)  Age: 18-65 years  Severity: HAM-D-17 ≥ 18, inadequate response to AD | | AD | BRE (192)  PBO (202) | | 43.0  42.7 | 23.4  28.7 | 85.4  84.7 | 2  fixed dose |
| Hobart 2018b (24)  Multi  DB | 503 | 6 | Diagnosis: MDD (DSM-IV-TR)  Age: 18-65 years  Severity: MADRS total score of ≥ 26, inadequate response to AD | | AD | BRE (197)  QUE (100)  PBO (206) | | 43.6  44.6  41.8 | 35.0  34.0  27.7 | 90.4  90.0  90.3 | BRE: 2 – 3  QUE: 150-300 |
| Ionesc 2016 (25)  US  DB | 20 | 4 | Diagnosis: MDD (DSM-IV)  Age: NR  Severity: HAM-D ≥ 5, Anger/Hostility Scale of the Symptom Questionnaire ≥ 5, partial remission | | AD | ILO (20) ^d)^  PBO (20) ^d)^ | | 46.1  46.1 | 30.0  30.0 | 84.6  84.6 | 1 – 8 |
| Kamijima 2013 (26)  Japan  DB | 586 | 6 | Diagnosis: MDD (DSM-IV)  Age: 20-65 years  Severity: HAM-D-17 ≥ 18, inadequate response to AD | | AD | ARI 3-15 (194)  ARI 3 (197)  PBO (195) | | 38.1  39.2  38.7 | 52.1  62.9  59.0 | NR | ARI 3 – 15: 9.8, and 3 fixed dose |
| Kamijima 2018 (27)  Multi  DB | 412 | 6 | Diagnosis: MDD (DSM-5)  Age: 20–65 years  Severity: HAM-D-17 ≥ 18, inadequate response to AD | | Sertraline | ARI (209)  PBO (203) | | 38.3  39.5 | 62.0  64.5 | 1.0  2.0 | 6.3 |
| Keitner 2009 (28)  US  DB | 97 | 4 | Diagnosis: MDD (DSM-IV)  Age: 18-65 years  Severity: MADRS ≥ 15, failed to respond or only partially responded to AD | | AD | RIS (63)  PBO (34) | | 45.5  44.6 | 42.2  45.5 | 93.7  84.8 | 1.6 |
| Lenze 2015 (29)  US and Canada  DB | 181 | 12 | Diagnosis: MDD (DSM)  Age: ≥ 60 years  Severity: MADRS ≥ 15, not achieve remission with venlafaxine monotherapy | | Venlafaxine | ARI (91)  PBO (90) | | 66.0  65.7 | 43  43 | 88  88 | Median=7 in remitters; Median=10 in non-remitters |
| Li 2016 (30)  US  DB | 23 | 8 | Diagnosis: MDD (DSM-IV)  Age: 18-65 years  Severity: HAM-D-17 ≥ 18, HAM-A ≥ 18 | | AD ^e)^ | QUE (11) ^f)^  PBO (12) ^f)^ | | 48.7  52.7 | 27.3  25.0 | 27.3  75.0 | 154 |
| Lin 2011 (31)  Taiwan  DB | 41 | 10 | Diagnosis: MDD (DSM-IV)  Age: 18-65 years  Severity: HAM-D-17 ≥ 14, HAM-D Item 3 ≤ 3 | | Sertraline | ARI (21)  PBO (20) | | 35.9  35.1 | 19.1  20.0 | NR | 2.5 |
| Mahmoud 2007 (32)  US  DB | 274 | 6 | Diagnosis: MDD (DSM-IV)  Age: 18-85 years  Severity: CGI-S score ≥ 4 | | AD | RIS (141)  PBO (133) | | 45.9  46.4 | 29.2  23.7 | 76.6  76.3 | 1 – 2 |
| Marcus 2008 (33)  US  DB | 381 | 6 | Diagnosis: MDD (DSM-IV)  Age: 18-65 years  Severity: HAM-D-17 ≥ 18, inadequate response to AD | | AD ^g)^ | ARI (191)  PBO (190) | | 44.6  44.4 | 34.0  32.6 | 89.0  88.9 | 11.0 |
| McIntyre 2007 (34)  Canada  DB | 58 | 8 | Diagnosis: MDD (DSM-IV)  Age: 18-65 years  Severity: HAM-D-17 ≥ 20, CGI-S ≥ 4, HAM-A ≥ 14 | | SSRI or venlafaxine | QUE (29)  PBO (29) | | 44  45 | 35  41 | NR | 182 |
| Papakostas 2015 (35)  US  DB | 139 | 8 | Diagnosis: MDD (DSM-IV)  Age: 18–65 years  Severity: QIDS-SR ≥ 10 | | Escitalopram | ZIP (71)  PBO (68) | | 44.7  44.2 | 31.0  27.9 | NR | 98 |
| Parker 2005 (36)  Australia  SB | 20 | 2 | Diagnosis: MDD (DSM-IV)  Age: NR  Severity: NR | | AD | OLA (10)  PBO (10) | | NR | 35.0 | NR | 2.5 – 5.0 |
| Quante 2013 (37)  Germany  DB | 36 | 6 | Diagnosis: MDD (DSM-IV)  Age: 18-65 years  Severity: HAM-D item 13 ≥ 2 and HSCL/SCL-90 ≥ 1 SD higher than the mean value of healthy controls | | Citalopram | QUE (19)  PBO (17) | | 45.1  48.4 | NR | NR | 310 |
| Reeves 2008 (38)  US  DB | 23 | 8 | Diagnosis: MDD (DSM-IV)  Age: 19-60 years  Severity: MADRS ≥ 25, MADRS suicidal subscore ≥ 4, with suicidal ideation despite treatment with AD | | AD | RIS (12)  PBO (11) | | 46.5  41.3 | 8.3  54.5 | NR | 1.17 |
| Sim 1978 (39)  NR  DB | 66 | 6 | Diagnosis: Endogenous depression  Age: 18-70 years  Severity: HAM-D ≥ 20 | | Imipramine | OXY 30 (16)  OXY 60 (18)  PBO (32) | | 43  51  50 | 12.5  27.8  31.3 | NR | 30 and 60 fixed dose |
| Stabl 1995 (40)  Switzerland and Austria  DB | 78 | 4 | Diagnosis: MDD (DSM-III-R)  Age: 18-70 years  Severity: HAM-D-17 ≥ 20, depression is rated as marked or severe on the CGI, and refractory to at least two previous treatments | | Moclobemide | THI (38)  PBO (40) | | 51  53 | 47.4  40.0 | NR | 100  fixed dose |
| Thase 2015a (41)  Multi  DB | 379 | 6 | Diagnosis: MDD (DSM-IV-TR)  Age: 18-65 years  Severity: HAM-D-17 ≥ 18, inadequate response to AD | | AD | BRE 2 (188)  PBO (191) | | 44.1  45.2 | 30.9  28.3 | 86.7  86.9 | 2  fixed dose |
| Thase 2015b (42)  Multi  DB | 677 | 6 | Diagnosis: MDD (DSM-IV-TR)  Age: 18-65 years  Severity: HAM-D-17 ≥ 18, inadequate response to AD | | AD | BRE 1 (226)  BRE 3 (230)  PBO (221) | | 45.7  44.5  46.6 | 30.1  32.2  33.9 | 81.0  87.4  85.1 | 1 and 3  fixed dose |
| Wade 2011 (43)  UK  DB | 165 | 8 | Diagnosis: MDD (DSM-IV)  Age: NR  Severity: HAM-D-17 ≥ 18, CGI-S ≥ 4 | | Citalopram | PIP (83)  PBO (82) | | 40.1  39.7 | 15.7  24.4 | 98.8  100 | 10  fixed dose |
| NCT00797966 (44)  US  DB | 429 | 6 | Diagnosis: MDD (DSM-IV-TR)  Age: NR  Severity: HAM-D-17 ≥ 18, inadequate response to AD | | AD | [BRE 0.15 (62)]  [BRE 0.5±0.25 (120)]  BRE 1.5±0.5 (121)  PBO (126) | | 43.9  44.0  43.7  43.3 | 33.9  28.3  33.9  34.9 | NR | 0.15  fixed dose  0.5±0.25  1.5±0.5 |
| NCT01052077 (45)  US  DB | 372 | 6 | Diagnosis: MDD (DSM-IV-TR)  Age: NR  Severity: HAM-D-17 ≥ 18, inadequate response to AD | | AD | BRE (185)  PBO (187) | | 44.7  42.7 | 32.0  33.5 | 75.5  67.5 | 1 – 3 |
| **- Number of studies:** 32 studies (32 reports)  **- Number of patients:** 8,349  [median=203 (range = 20 − 819)] **- Country:**  US (studies=15, n=3,315)  Multinational (studies=10, n=4,062) Australia (study=1, n=20)  Canada (study=1, n=58)  Germany (study=1, n=36)  Japan (study=1, n=586)  NR (study=1, n=66)  Taiwan (study=1, n=41)  UK (study=1, n=165) | | | | **- Blinding status:** DB (studies=30, n=8,268), SB (study=1, n=20), OL (study=1, n=61)  **- Mean duration:** 7.4 (range **=**2 − 36)  **- Antipsychotics:**  ARI (studies=8, n=2,537), BRE (study=6, n=2,754),  CAR (study=1, n=819), ILO (study=1, n=20),  OLA (study=2, n=146), OXY (study=1, n=66),  PIP (study=1, n=165), QUE (studies=6, n=1,056),  RIS (studies=3, n=394), THI (study=1, n=78),  ZIP (study=2, n=200) | | | **- Age:** 45.4±5.2 (range=35.1 – 66) years  **- Gender** (Male 34.2%)  **- Race** (White 82.1%) | | | | |

**Notes:**

[drug groups or numbers in squared brackets were not used in any analysis, neither the primary one nor in a sensitivity analysis]

**Abbreviations:**

AD=antidepressant, AMI=amisulpride, AMIN=amineptine, ARI=aripiprazole, BRE=brexpiprazole, CAR=cariprazine, CGI-S=Clinical Global Impressions－Severity of illness scale, DSM-IV-TR=Diagnostic and Statistical Manual of Mental Disorders, Fourth Edition. Text Revision, ECT=Electro Convulsive Therapy, ESC=escitalopram, FDA=Food and Drug Administration, FLU=flupenthixol, HAL=haloperidol, HAM-A= Hamilton Anxiety Rating Scale, HAM-D=Hamilton Rating Scale for Depression, HSCL/SCL-90=Hopkins Check List / Symptoms Check List 90, ILO=iloperidone, MADRS=Montgomery Åsberg Depression Rating Scale, MDD=Major Depressive Disorder, MMSE=Mini-Mental State Examination, OLA=olanzapine, OFC=olanzapine/fluoxetine combination, OXY=oxypertine, PBO=placebo, PIP=pipamperone, QIDS-SR=Quick Inventory of Depressive Symptomatology-Self-Rated, QUE=quetiapine, RIS=risperidone, SD=standard deviation, SNRI=Serotonin & Norepinephrine Reuptake Inhibitor, SSRI=Selective Serotonin Reuptake Inhibitor, SUL=sulpiride, THI=thioridazine, XR= extended release, ZIP=ziprasidone

1. One hundred twenty outpatients were enrolled in a 12-week study that was divided into two 6-week periods according to the sequential parallel comparison design. Patients were randomized in a 2:3:3 fashion to receive ziprasidone for 12 weeks, placebo for 6 weeks followed by ziprasidone for 6 weeks, or placebo for 12 weeks.
2. Two hundred twenty five MDD subjects were randomized to adjunctive treatment with aripiprazole 2 mg/day or placebo across two 30-day phases, with a 2: 3:3 randomization ratio to drug/drug (aripiprazole 2 mg/day in phase 1; 5 mg/day in phase 2), placebo/placebo (placebo in both phases), and placebo/drug (placebo in phase 1; aripiprazole 2 mg/day in phase 2).
3. Remission was defined as the absence of delusions and hallucinations and a 17-item HAM-D score of 10 or less for 2 consecutive weeks. In addition, participants who met criteria for “near remission” following 12weeks of acute treatment were also eligible to enter the stabilization phase. Near remission was defined as the absence of delusions and hallucinations, an HAM-D score of 11 to 15 with 50% or more reduction in baseline HAM-D score, and being rated as “very much improved” or “much improved” on the Clinical Global Impression scale.
4. Crossover study
5. SSRI or SNRI
6. Monotherapy or adjunctive therapy to antidepressant(s)
7. Escitalopram, fluoxetine, paroxetine controlled-release, sertraline, or venlafaxine extended-release

**Table S3 Key findings**

1. Nelson JC, Papakostas GI. Atypical antipsychotic augmentation in major depressive disorder: a meta-analysis of placebo-controlled randomized trials. The American journal of psychiatry. 2009;166(9):980-91.
2. Spielmans GI, Berman MI, Linardatos E, Rosenlicht NZ, Perry A, Tsai AC. Adjunctive atypical antipsychotic treatment for major depressive disorder: a meta-analysis of depression, quality of life, and safety outcomes. PLoS medicine. 2013;10(3):e1001403.

**Abbreviations:**

AE=adverse event, AMI=amisulpride, AP=antipsychotic drug, ARI=aripiprazole, BRE=brexpiprazole, CAR=cariprazine, CI=confidence interval, FLU= flupenthixol, HAL=haloperidol, ILO=iloperidone, MDD=major depressive disorder, NR=not reported, OLA=olanzapine, OR=odds ratio, OXY=oxypertine, PIP=pipamperone, QUE=quetiapine, RIS=risperidone, RR=risk ratio, SUL=sulpiride, THI=thioridazine, ZIP=ziprasidone

**Table S4 Results of meta-analysis for secondary outcomes**

| **Category** | **Subcategory** | **Specific Outcomes** | **Medication** | **N** | **n** | **RR/**  ***SMD*** | **95% CI** | | **Results: P-value** | **Heterogeneity** | | **NNH ^a)^**  **(95% CI)** |
| --- | --- | --- | --- | --- | --- | --- | --- | --- | --- | --- | --- | --- |
|  |  |  |  |  |  |  | **Lower limit** | **Upper limit** |  | **P-value** | **I^2^** |  |
| ***Monotherapy*** | | | | | | | | | | | | |
| Efficacy | *Anxiety symptom scale* |  | *QUE* | *2* | *642* | *-0.37* | *-0.78* | *0.04* | *0.076* | ***0.009*** | ***85.5*** | *−* |
|  | CGI-I=1 or 2 (very much or much improved) |  | **QUE** | **5** | **2,087** | **1.35** | **1.12** | **1.62** | **0.002** | 0.002 | 75.8 | **7 (4, 19) ^b)^** |
|  |  |  | **SUL** | **1** | **155** | **1.28** | **1.00** | **1.64** | **0.046** | − | − | **7 (4, 151) ^b)^** |
|  | *CGI-S* |  | ***QUE*** | ***3*** | ***1,413*** | ***-0.35*** | ***-0.63*** | ***-0.07*** | ***0.015*** | ***0.002*** | ***84.1*** | *−* |
|  |  |  | *ZIP* | *2* | *166* | *0.16* | *-0.21* | *0.54* | *0.389* | *0.281* | *14.1* | *−* |
|  | *SDS* |  | ***QUE*** | ***1*** | ***771*** | ***-0.17*** | ***-0.31*** | ***-0.03*** | ***0.018*** | *−* | *−* | *−* |
|  | *SDS Family life* |  | ***QUE*** | ***1*** | ***771*** | ***-0.17*** | ***-0.31*** | ***-0.03*** | ***0.018*** | *−* | *−* | *−* |
|  | *SDS Social life* |  | ***QUE*** | ***1*** | ***771*** | ***-0.17*** | ***-0.31*** | ***-0.03*** | ***0.018*** | *−* | *−* | *−* |
|  | *SDS Work/School* |  | *QUE* | *1* | *771* | *-0.09* | *-0.23* | *0.05* | *0.202* | *−* | *−* | *−* |
| Safety | SAE |  | AMI | 1 | 145 | 0.49 | 0.09 | 2.61 | 0.406 | − | − | NA |
|  |  |  | QUE | 5 | 2481 | 1.28 | 0.66 | 2.48 | 0.459 | 0.562 | 0.0 | NA |
|  |  |  | SUL | 1 | 171 | 0.21 | 0.01 | 4.35 | 0.314 | − | − | NA |
|  |  |  | ZIP | 1 | 205 | 0.52 | 0.02 | 12.6 | 0.686 | − | − | NA |
|  | Anticholinergic AEs | Blurred vision | AMI | 1 | 145 | 2.63 | 0.73 | 9.52 | 0.141 | − | − | NA |
|  |  |  | QUE | 1 | 461 | 2.75 | 0.81 | 9.31 | 0.103 | − | − | **30 (15, 1894)** |
|  |  |  | ZIP | 1 | 205 | 1.56 | 0.22 | 10.9 | 0.652 | − | − | NA |
|  |  | Dry mouth | AMI | 1 | 145 | 0.99 | 0.52 | 1.87 | 0.966 | − | − | NA |
|  |  |  | **QUE** | **8** | **3271** | **3.21** | **2.40** | **4.29** | **<0.001** | 0.103 | 41.3 | **7 (5, 11)** |
|  |  |  | SUL | 1 | 171 | 0.71 | 0.12 | 4.12 | 0.700 | − | − | NA |
|  |  |  | ZIP | 1 | 205 | 1.56 | 0.52 | 4.68 | 0.425 | − | − | NA |
|  | Central nervous system | Anxiety | AMI | 1 | 212 | 0.52 | 0.10 | 2.77 | 0.443 | − | − | NA |
|  |  |  | QUE | 1 | 312 | 2.96 | 0.98 | 8.98 | 0.055 | − | − | **20 (11, 466)** |
|  |  | Dizziness | AMI | 1 | 145 | 0.52 | 0.26 | 1.04 | 0.064 | − | − | NA |
|  |  |  | **QUE** | **7** | **3249** | **1.62** | **1.32** | **1.99** | **<0.001** | 0.489 | 0.0 | **19 (12, 35)** |
|  |  |  | SUL | 1 | 171 | 4.24 | 0.48 | 37.2 | 0.192 | − | − | NA |
|  |  |  | ZIP | 1 | 205 | 1.56 | 0.22 | 10.9 | 0.652 | − | − | NA |
|  |  | Fatigue | **QUE** | **7** | **3249** | **1.97** | **1.38** | **2.82** | **<0.001** | 0.509 | 0.0 | **36 (19, 91)** |
|  |  |  | SUL | 1 | 171 | 2.65 | 0.53 | 13.3 | 0.236 | − | − | NA |
|  |  | Headache | AMI | 1 | 212 | 1.04 | 0.35 | 3.12 | 0.946 | − | − | NA |
|  |  |  | HAL | 1 | 53 | 0.85 | 0.42 | 1.71 | 0.647 | − | − | NA |
|  |  |  | QUE | 8 | 3271 | 0.96 | 0.78 | 1.19 | 0.727 | 0.184 | 30.5 | NA |
|  |  |  | SUL | 1 | 171 | 0.53 | 0.14 | 2.05 | 0.358 | − | − | NA |
|  |  |  | ZIP | 1 | 205 | 2.34 | 0.40 | 13.7 | 0.345 | − | − | NA |
|  | Arousal-related AEs | Irritability | QUE | 4 | 2,266 | 0.82 | 0.44 | 1.54 | 0.542 | 0.164 | 41.2 | NA |
|  | Cardiovascular AEs | Palpitations | AMI | 2 | 357 | 0.93 | 0.47 | 1.83 | 0.828 | 0.876 | 0.0 | NA |
|  |  |  | QUE | 1 | 312 | 0.99 | 0.33 | 2.99 | 0.982 | − | − | NA |
|  | Gastrointestinal AEs | Constipation | AMI | 1 | 145 | 0.78 | 0.43 | 1.41 | 0.409 | − | − | NA |
|  |  |  | **QUE** | **7** | **3249** | **2.30** | **1.56** | **3.41** | **<0.001** | 0.404 | 2.8 | **32 (18, 75)** |
|  |  |  | SUL | 1 | 171 | 0.21 | 0.01 | 4.35 | 0.314 | − | − | NA |
|  |  |  | ZIP | 1 | 205 | 1.56 | 0.32 | 7.55 | 0.579 | − | − | NA |
|  |  | Diarrhea | QUE | 6 | 2934 | 0.82 | 0.60 | 1.11 | 0.196 | 0.324 | 14.0 | NA |
|  |  |  | SUL | 1 | 171 | 0.21 | 0.01 | 4.35 | 0.314 | − | − | NA |
|  |  | Dyspepsia | QUE | 3 | 1490 | 1.34 | 0.79 | 2.29 | 0.282 | 0.984 | 0.0 | NA |
|  |  | Nausea | AMI | 1 | 212 | 0.69 | 0.12 | 4.06 | 0.684 | − | − | NA |
|  |  |  | QUE | 8 | 3,271 | 0.89 | 0.63 | 1.26 | 0.521 | 0.090 | 43.3 | NA |
|  |  |  | SUL | 1 | 171 | 0.35 | 0.01 | 8.55 | 0.522 | − | − | NA |
|  |  |  | ZIP | 1 | 205 | 0.78 | 0.07 | 8.48 | 0.839 | − | − | NA |
|  | Other AEs | Nasopharyngitis | QUE | 3 | 1,395 | 0.53 | 0.19 | 1.48 | 0.226 | **0.048** | **67.0** | NA |
| ***Adjunctive therapy*** | | | | | | | | | | | | |
| Efficacy | *Anxiety symptom scale* |  | ***BRE*** | ***2*** | ***769*** | ***-0.19*** | ***-0.33*** | ***-0.05*** | ***0.010*** | *0.998* | *0.0* | − |
|  |  |  | ***QUE*** | ***2*** | ***80*** | ***-0.70*** | ***-1.18*** | ***-0.22*** | ***0.005*** | *0.295* | *8.7* | − |
|  |  |  | ***ZIP*** | ***2*** | ***193*** | ***-0.29*** | ***-0.58*** | ***-0.01*** | ***0.046*** | *0.466* | *0.0* | − |
|  | CGI-I=1 or 2 (very much or much improved) |  | **ARI** | **4** | **1693** | **1.41** | **1.26** | **1.57** | **<0.001** | 0.973 | 0.0 | **7 (5, 10) ^b)^** |
|  |  |  | **BRE** | **3** | **1002** | **1.17** | **1.03** | **1.34** | **0.016** | 0.795 | 0.0 | **14 (7, 75) ^b)^** |
|  |  |  | **QUE** | **2** | **919** | **1.22** | **1.08** | **1.39** | **0.002** | 0.859 | 0.0 | **9 (6, 27) ^b)^** |
|  |  |  | THI | 1 | 78 | 1.05 | 0.81 | 1.37 | 0.699 | − | − | NA |
|  | *CGI-S* |  | ***ARI*** | ***8*** | ***2404*** | ***-0.26*** | ***-0.37*** | ***-0.16*** | ***<0.001*** | *0.164* | *33.0* | *−* |
|  |  |  | ***BRE*** | ***6*** | ***2167*** | ***-0.20*** | ***-0.29*** | ***-0.10*** | ***<0.001*** | *0.256* | *23.7* | *−* |
|  |  |  | *CAR* | *1* | *808* | *-0.09* | *-0.24* | *0.06* | *0.223* | *−* | *−* | *−* |
|  |  |  | *QUE* | *2* | *340* | *0.01* | *-0.22* | *0.23* | *0.942* | *0.843* | *0.0* | *−* |
|  |  |  | ***RIS*** | ***3*** | ***376*** | ***-1.06*** | ***-1.80*** | ***-0.32*** | ***0.005*** | ***0.001*** | ***85.4*** | *−* |
|  |  |  | ***ZIP*** | ***2*** | ***199*** | ***-0.37*** | ***-0.65*** | ***-0.08*** | ***0.012*** | *0.757* | *0.0* | *−* |
|  | *SDS* |  | ***ARI*** | ***3*** | ***1253*** | ***-0.27*** | ***-0.38*** | ***-0.15*** | ***<0.001*** | *0.419* | *0.0* | *−* |
|  |  |  | ***BRE*** | ***5*** | ***1744*** | ***-0.20*** | ***-0.29*** | ***-0.11*** | ***<0.001*** | *0.774* | *0.0* | *−* |
|  |  |  | ***CAR*** | ***1*** | ***808*** | ***-0.15*** | ***-0.30*** | ***-0.01*** | ***0.043*** | *−* | *−* | *−* |
|  |  |  | *QUE* | *2* | *326* | *0.23* | *0.00* | *0.46* | *0.051* | *0.629* | *0.0* | *−* |
|  |  |  | ***RIS*** | ***1*** | ***223*** | ***-0.57*** | ***-0.84*** | ***-0.30*** | ***<0.001*** | *−* | *−* | *−* |
|  | *SDS Family life* |  | ***ARI*** | ***3*** | ***1256*** | ***-0.34*** | ***-0.46*** | ***-0.23*** | ***<0.001*** | *0.379* | *0.0* | *−* |
|  |  |  | ***BRE*** | ***3*** | ***1165*** | ***-0.23*** | ***-0.35*** | ***-0.12*** | ***<0.001*** | *0.967* | *0.0* | *−* |
|  |  |  | ***QUE*** | ***1*** | ***304*** | ***0.25*** | ***0.01*** | ***0.49*** | ***0.044*** | *−* | *−* | *−* |
|  |  |  | ***RIS*** | ***1*** | ***223*** | ***-0.39*** | ***-0.66*** | ***-0.13*** | ***0.004*** | *−* | *−* | *−* |
|  | *SDS Social life* |  | ***ARI*** | ***3*** | ***1,256*** | ***-0.31*** | ***-0.43*** | ***-0.20*** | ***<0.001*** | *0.398* | *0.0* | *−* |
|  |  |  | ***BRE*** | ***3*** | ***1,165*** | ***-0.21*** | ***-0.33*** | ***-0.10*** | ***<0.001*** | *0.996* | *0.0* | *−* |
|  |  |  | ***QUE*** | ***1*** | ***304*** | ***0.25*** | ***0.01*** | ***0.49*** | ***0.044*** | *−* | *−* | *−* |
|  |  |  | ***RIS*** | ***1*** | ***223*** | ***-0.55*** | ***-0.81*** | ***-0.28*** | ***<0.001*** | *−* | *−* | *−* |
|  | *SDS Work/School* |  | *ARI* | *3* | *1,104* | *-0.17* | *-0.42* | *0.08* | *0.176* | ***0.020*** | ***74.5*** | *−* |
|  |  |  | *BRE* | *3* | *1,030* | *-0.04* | *-0.17* | *0.08* | *0.482* | *0.678* | *0.0* | *−* |
|  |  |  | *QUE* | *1* | *203* | *0.24* | *-0.05* | *0.53* | *0.111* | *−* | *−* | *−* |
| Safety | SAE |  | ARI | 6 | 2,264 | 0.96 | 0.43 | 2.17 | 0.924 | 0.775 | 0.0 | NA |
|  |  |  | BRE | 4 | 1,447 | 1.02 | 0.30 | 3.54 | 0.971 | 0.488 | 0.0 | NA |
|  |  |  | CAR | 1 | 812 | 0.97 | 0.09 | 10.7 | 0.983 | − | − | NA |
|  |  |  | ILO | 1 | 26 | 1.00 | 0.16 | 6.07 | 1.000 | − | − | NA |
|  |  |  | OLA | 1 | 126 | 0.97 | 0.47 | 1.99 | 0.931 | − | − | NA |
|  |  |  | PIP | 1 | 163 | 0.32 | 0.01 | 7.78 | 0.485 | − | − | NA |
|  |  |  | QUE | 2 | 936 | 0.73 | 0.21 | 2.59 | 0.631 | 0.757 | 0.0 | NA |
|  |  |  | RIS | 1 | 268 | 0.19 | 0.01 | 3.95 | 0.284 | − | − | NA |
|  |  |  | ZIP | 1 | 139 | 0.96 | 0.14 | 6.61 | 0.965 | − | − | NA |
|  | EPS | Dystonia | ARI | 1 | 586 | 10.5 | 0.62 | 178 | 0.104 | − | − | **40 (24, 124)** |
|  |  |  | QUE | 1 | 58 | 3.00 | 0.33 | 27.2 | 0.329 | − | − | NA |
|  |  |  | RIS | 1 | 268 | 0.32 | 0.01 | 7.76 | 0.483 | − | − | NA |
|  | Anticholinergic AEs | Blurred vision | **ARI** | **2** | **706** | **4.05** | **1.68** | **9.75** | **0.002** | 0.920 | 0.0 | **19 (7, 86)** |
|  |  |  | QUE | 1 | 23 | 3.25 | 0.15 | 72.4 | 0.457 | − | − | NA |
|  |  | Dry mouth | ARI | 2 | 944 | 1.38 | 0.20 | 9.58 | 0.747 | **0.012** | **84.3** | NA |
|  |  |  | BRE | 1 | 403 | 2.09 | 0.19 | 22.9 | 0.546 | − | − | NA |
|  |  |  | CAR | 1 | 812 | 1.67 | 0.73 | 3.83 | 0.225 | − | − | NA |
|  |  |  | ILO | 1 | 26 | 6.00 | 0.83 | 43.1 | 0.075 | − | − | **3 (2, 13)** |
|  |  |  | OLA | 1 | 126 | 0.32 | 0.01 | 7.78 | 0.486 | − | − | NA |
|  |  |  | PIP | 1 | 163 | 1.93 | 0.76 | 4.89 | 0.167 | − | − | NA |
|  |  |  | **QUE** | **6** | **1437** | **3.87** | **2.70** | **5.54** | **<0.001** | 0.383 | 5.3 | **7 (4, 11)** |
|  |  |  | RIS | **3** | **388** | **6.76** | **1.87** | **24.41** | **0.004** | 0.821 | 0.0 | **16**  **(4, 101)** |
|  |  |  | THI | 1 | 78 | 7.36 | 0.39 | 138 | 0.182 | − | − | NA |
|  |  |  | ZIP | 2 | 200 | 1.34 | 0.15 | 11.9 | 0.794 | 0.118 | 59.2 | NA |
|  | Central nervous system | Anxiety | BRE | 4 | 1625 | 2.33 | 0.97 | 5.63 | 0.060 | 0.518 | 0.0 | NA |
|  |  |  | QUE | 4 | 501 | 0.58 | 0.20 | 1.72 | 0.329 | 0.355 | 3.4 | NA |
|  |  |  | ZIP | 1 | 139 | 8.62 | 0.47 | 157 | 0.146 | − | − | NA |
|  |  | Dizziness | ARI | 4 | 1387 | 0.96 | 0.55 | 1.68 | 0.885 | 0.470 | 0.0 | NA |
|  |  |  | CAR | 1 | 812 | 2.34 | 0.90 | 6.06 | 0.080 | − | − | NA |
|  |  |  | ILO | 1 | 26 | 5.00 | 0.26 | 95.0 | 0.284 | − | − | NA |
|  |  |  | OLA | 1 | 126 | 2.42 | 0.49 | 12.0 | 0.279 | − | − | NA |
|  |  |  | PIP | 1 | 163 | 1.29 | 0.47 | 3.54 | 0.627 | − | − | NA |
|  |  |  | QUE | 4 | 1017 | 1.43 | 0.88 | 2.31 | 0.144 | 0.309 | 16.5 | NA |
|  |  |  | RIS | 2 | 291 | 1.66 | 0.50 | 5.48 | 0.408 | 0.918 | 0.0 | NA |
|  |  |  | ZIP | 2 | 200 | 2.32 | 0.59 | 9.12 | 0.227 | 0.295 | 8.8 | NA |
|  |  | Fatigue | **ARI** | **4** | **1126** | **2.07** | **1.28** | **3.36** | **0.003** | 0.870 | 0.0 | **23 (11, 87)** |
|  |  |  | **BRE** | **5** | **1997** | **2.10** | **1.17** | **3.77** | **0.013** | 0.642 | 0.0 | **58 (23, 376)** |
|  |  |  | **CAR** | **1** | **812** | **1.95** | **1.02** | **3.71** | **0.042** | − | − | **26 (14, 163)** |
|  |  |  | OLA | 1 | 126 | 1.94 | 0.18 | 20.8 | 0.585 | − | − | NA |
|  |  |  | PIP | 1 | 163 | 1.08 | 0.44 | 2.67 | 0.860 | − | − | NA |
|  |  |  | **QUE** | **5 ^c)^** | **1,379** | **2.65** | **1.60** | **4.38** | **<0.001** | 0.430 | 0.0 | **13 (7, 35)** |
|  |  |  | RIS | 2 | 365 | 1.07 | 0.01 | 98.2 | 0.976 | **0.030** | **78.7** | NA |
|  |  | Headache | **ARI** | **6** | **2429** | **0.69** | **0.51** | **0.95** | **0.021** | 0.538 | 0.0 | **-45 ^d)^ (-261, -28)** |
|  |  |  | **BRE** | **5** | **1,865** | **0.69** | **0.48** | **0.99** | **0.042** | 0.601 | 0.0 | **-43 ^d)^ (-1007, -26)** |
|  |  |  | ILO | 1 | 26 | 2.00 | 0.21 | 19.4 | 0.550 | − | − | NA |
|  |  |  | OLA | 1 | 126 | 2.91 | 0.31 | 27.2 | 0.350 | − | − | NA |
|  |  |  | PIP | 1 | 163 | 1.07 | 0.62 | 1.83 | 0.818 | − | − | NA |
|  |  |  | QUE | 6 | 1437 | 0.73 | 0.50 | 1.07 | 0.109 | 0.353 | 9.8 | NA |
|  |  |  | RIS | **3** | **388** | **0.46** | **0.24** | **0.89** | **0.020** | 0.227 | 32.5 | **-10 ^d)^ (-44, -7)** |
|  |  |  | ZIP | 2 | 200 | 1.09 | 0.19 | 6.43 | 0.923 | 0.110 | 60.9 | NA |
|  | CNS | Irritability | ILO | 1 | 26 | 3.00 | 0.13 | 67.5 | 0.489 | − | − | NA |
|  |  |  | QUE | 2 | 468 | 0.85 | 0.39 | 1.87 | 0.686 | 0.381 | 0.0 | NA |
|  |  |  | ZIP | 1 | 139 | 6.70 | 0.85 | 53.1 | 0.071 | − | − | **12 (7, 113)** |
|  | Cardiovascular AEs | Palpitations | ARI | 1 | 41 | 0.32 | 0.01 | 7.38 | 0.475 | − | − | NA |
|  |  |  | ILO | 1 | 26 | 5.00 | 0.26 | 95.0 | 0.284 | − | − | NA |
|  | Gastrointestinal AEs | Constipation | **ARI** | **5** | **2071** | **2.19** | **1.33** | **3.61** | **0.002** | 0.536 | 0.0 | **38 (18, 138)** |
|  |  |  | BRE | 1 | 372 | 2.78 | 0.90 | 8.57 | 0.075 | − | − | NA |
|  |  |  | CAR | 1 | 812 | 1.95 | 0.74 | 5.14 | 0.177 | − | − | NA |
|  |  |  | OLA | 1 | 126 | 0.32 | 0.01 | 7.78 | 0.486 | − | − | NA |
|  |  |  | **QUE** | **3** | **994** | **2.42** | **1.30** | **4.51** | **0.005** | 0.582 | 0.0 | **22 (9, 104)** |
|  |  |  | RIS | 2 | 365 | 1.47 | 0.57 | 3.76 | 0.423 | 0.878 | 0.0 | NA |
|  |  |  | ZIP | 1 | 61 | 4.50 | 0.25 | 79.7 | 0.305 | − | − | NA |
|  |  | Diarrhea | ARI | 5 | 2050 | 0.71 | 0.46 | 1.08 | 0.112 | 0.751 | 0.0 | NA |
|  |  |  | BRE | 3 | 998 | 1.02 | 0.49 | 2.16 | 0.950 | 0.176 | 42.4 | NA |
|  |  |  | ILO | 1 | 26 | 3.00 | 0.13 | 67.5 | 0.489 | − | − | NA |
|  |  |  | OLA | 1 | 126 | 3.88 | 0.45 | 33.7 | 0.220 | − | − | NA |
|  |  |  | PIP | 1 | 163 | 0.74 | 0.34 | 1.59 | 0.443 | − | − | NA |
|  |  |  | QUE | 1 | 445 | 1.00 | 0.48 | 2.07 | 0.993 | − | − | NA |
|  |  |  | RIS | 2 | 291 | 0.59 | 0.21 | 1.70 | 0.327 | 0.954 | 0.0 | NA |
|  |  |  | ZIP | 1 | 139 | 0.55 | 0.17 | 1.79 | 0.318 | − | − | NA |
|  |  | Dyspepsia | RIS | 1 | 268 | 0.72 | 0.16 | 3.14 | 0.659 | − | − | NA |
|  |  | Nausea | ARI | 7 | 2470 | 0.87 | 0.62 | 1.22 | 0.432 | 0.738 | 0.0 | NA |
|  |  |  | BRE | 1 | 247 | 0.69 | 0.12 | 4.08 | 0.686 | − | − | NA |
|  |  |  | **CAR** | **1** | **812** | **2.02** | **1.12** | **3.64** | **0.019** | − | − | **20 (12, 72)** |
|  |  |  | ILO | 1 | 26 | 2.00 | 0.21 | 19.4 | 0.550 | − | − | NA |
|  |  |  | OLA | 1 | 126 | 0.48 | 0.09 | 2.55 | 0.392 | − | − | NA |
|  |  |  | PIP | 1 | 163 | 0.70 | 0.42 | 1.17 | 0.174 | − | − | NA |
|  |  |  | QUE | 3 | 994 | 0.98 | 0.61 | 1.58 | 0.928 | 0.530 | 0.0 | NA |
|  |  |  | RIS | 2 | 291 | 0.44 | 0.14 | 1.35 | 0.152 | 0.570 | 0.0 | NA |
|  |  |  | ZIP | 2 | 200 | 0.98 | 0.06 | 15.1 | 0.991 | 0.073 | 68.8 | NA |
|  | Other AEs | Nasopharyngitis | ARI | 2 | 998 | 1.12 | 0.81 | 1.55 | 0.481 | 0.802 | 0.0 | NA |
|  |  |  | BRE | 4 | 1,462 | 1.57 | 0.91 | 2.72 | 0.108 | 0.502 | 0.0 | NA |
|  |  |  | PIP | 1 | 163 | 0.69 | 0.23 | 2.08 | 0.508 | − | − | NA |
|  |  |  | QUE | 1 | 491 | 0.49 | 0.21 | 1.15 | 0.100 | − | − | NA |

**Notes:**

Significant (P<.05) results are in bold. Continuous data (SMD) in italics.

RR values >1 indicate superiority of antipsychotics compared to placebo for positive outcomes, while RR values >1 indicate inferiority of antipsychotics compared to placebo for negative outcomes.

SMDs <0 indicate superiority of antipsychotics compared to placebo in symptom scale score. SMDs >0 indicate that antipsychotics had higher laboratory values than placebo.

1. NNHs for individual adverse event were calculated.
2. NNTs were calculated for positive outcome.
3. Includes a study (Hobart 2018) with an incidence risk of 0% in both antipsychotic and placebo arms.
4. Negative NNH indicates that the placebo was more harmful than the antipsychotics.
5. RR was not calculable as an incidence risk was 0% in both antipsychotic and placebo arms (Alexopoulos 2008).

**Abbreviations:**

AMI=amisulpride, ARI=aripiprazole, BRE=brexpiprazole, CAR=cariprazine, CGI-S= Clinical Global Impressions - severity of illness, CI=confidence interval, EPS=extrapyramidal symptoms, ILO=iloperidone, LUR=lurasidone, N=the number of studies, n=the number of patients, NA=not applicable, NNH=number needed to harm, OLA=olanzapine, PER=perphenazine, PIP=pipamperone, QUE=quetiapine, RIS=risperidone, RR=risk ratio, SAE=serious adverse event, SDS= the Sheehan Disability Scale, SMD=standardized mean difference, SUL=sulpiride, THI=thioridazine, ZIP=ziprasidone

**Table S5 Meta-regression analyses for co-primary outcomes**

| **Administration** | **Outcomes** | **Covariant** | **N** | **n** | **Coefficient** | **95% CI** | | **P-value** |
| --- | --- | --- | --- | --- | --- | --- | --- | --- |
|  |  |  |  |  |  | **Lower limit** | **Upper limit** |  |
| Monotherapy | Treatment response | **Mean age** | **10** | **2,733** | **0.015** | **0.005** | **0.024** | **0.002** |
|  |  | % Male | 10 | 2,733 | -0.007 | -0.025 | 0.011 | 0.464 |
|  |  | **% Caucasian** | **6** | **2,293** | **0.013** | **0.006** | **0.019** | **<0.001** |
|  |  | Publication year | 10 | 2,733 | -0.018 | -0.039 | 0.003 | 0.096 |
|  |  | Sample size | 10 | 2,733 | 0.000 | -0.001 | 0.001 | 0.814 |
|  |  | Trial duration | 10 | 2,733 | 0.027 | -0.001 | 0.055 | 0.058 |
|  |  | Illness duration | 4 | 1147 | 0.009 | -0.075 | 0.093 | .84 |
|  |  | # of lifetime DEP | 2 ^a)^ | NA | | | | |
|  |  | **# of DEP  in past year** | **4** | **1780** | **-0.203** | **-0.375** | **-0.030** | **.02** |
|  |  | DDD ratio | 13 | 3,241 | -0.250 | -0.657 | 0.157 | 0.228 |
|  | Discontinuation due to adverse event | Mean age | 11 | 3,640 | -0.003 | -0.037 | 0.031 | 0.867 |
|  |  | % Male | 11 | 3,640 | -0.002 | -0.055 | 0.051 | 0.940 |
|  |  | % Caucasian | 6 | 2,358 | -0.005 | -0.031 | 0.022 | 0.725 |
|  |  | Publication year | 12 | 3,693 | -0.004 | -0.084 | 0.077 | 0.932 |
|  |  | Sample size | 12 | 3,693 | -0.001 | -0.002 | 0.001 | 0.197 |
|  |  | Trial duration | 12 | 3,693 | -0.013 | -0.029 | 0.003 | 0.119 |
|  |  | Illness duration | 5 | 1950 | -0.088 | -0.281 | 0.106 | .37 |
|  |  | # of lifetime DEP | 2 ^a)^ | NA | | | | |
|  |  | # of DEP  in past year | 4 | 1832 | 0.090 | -0.763 | 0.943 | .84 |
|  |  | DDD ratio | 15 | 4,218 | -0.361 | -1.713 | 0.990 | 0.600 |
| Adjunctive therapy | Treatment response | **Mean age** | **28** | **7,366** | **-0.025** | **-0.047** | **-0.003** | **0.027** |
|  |  | % Male | 27 | 7,330 | 0.000 | -0.007 | 0.008 | 0.908 |
|  |  | % Caucasian | 20 | 6,196 | -0.001 | -0.005 | 0.002 | 0.413 |
|  |  | Publication year | 28 | 7,366 | 0.008 | -0.005 | 0.021 | 0.211 |
|  |  | Sample size | 28 | 7,366 | 0.000 | -0.001 | 0.000 | 0.729 |
|  |  | Trial duration | 28 | 7,366 | 0.034 | -0.037 | 0.105 | 0.349 |
|  |  | Illness duration | 2 ^a)^ | NA | | | | |
|  |  | **# of lifetime DEP** | **9** | **2,705** | **0.109** | **0.007** | **0.211** | **0.036** |
|  |  | # of DEP  in past year | 2 ^a)^ | NA | | | | |
|  |  | DDD ratio | 25 | 6,474 | 0.239 | -0.039 | 0.516 | 0.092 |
|  | Discontinuation due to adverse event | Mean age | 26 | 7,553 | -0.021 | -0.084 | 0.043 | 0.527 |
|  |  | % Male | 26 | 7,553 | -0.012 | -0.043 | 197.0 | 0.471 |
|  |  | % Caucasian | 20 | 6,404 | -0.002 | -0.019 | 0.015 | 0.807 |
|  |  | Publication year | 26 | 7,553 | 0.052 | -0.017 | 0.120 | 0.142 |
|  |  | **Sample size** | **26** | **7,553** | **0.001** | **0.000** | **0.003** | **0.017** |
|  |  | Trial duration | 26 | 7,553 | 0.065 | -0.117 | 0.248 | 0.482 |
|  |  | Illness duration | 3 ^a)^ | NA | | | | |
|  |  | # of lifetime DEP | 8 | 2,760 | 0.175 | -0.326 | 0.676 | .49 |
|  |  | # of DEP  in past year | 2 ^a)^ | NA | | | | |
|  |  | **DDD ratio** | **25** | **6,772** | **1.757** | **0.813** | **2.700** | **<0.001** |

**Notes:**Significant (P<0.05) results are in bold.

1. not enough studies for meta-regression analysis

**Abbreviations:**

CI=confidence interval, DDD=daily defined dose, DEP=depressive episodes, N=number of studies, n=number of patients, NA=not applicable

**Figure S1 Flow-diagram describing the search process**

4,377 records identified

through database searching

2,188 records excluded because they were clearly not relevant or duplicates

2,189 records screened

5 full-text articles excluded with reasons of no original data

43 full-text articles assessed for eligibility

**Included**

**Eligibility**

**Screening**

**Identification**

2,146 records excluded:

Not major depressive disorder: N=971

Review article: N=967

Not clinical trial: N=121

No antipsychotics: N=44

No available data or other reason: N=29

No placebo arm: N=11

Not human data: N=3

45 studies (45 articles) included in qualitative synthesis (meta-analysis)

Additional studies identified through other sources: N=7

**Figure S2 Meta-regression analysis in monotherapy**

1. **Association between treatment response and mean age**

Coefficient=0.0145, 95% CI=0.0052 − 0.0238, p=0.0022

**(B) Association between treatment response and % Caucasian**

Coefficient=0.0126, 95% CI=0.0064 – 0.0189, p=0.0001

**(C) Association between treatment response and number of depressive episodes in past year**

Coefficient=-0.2025, 95% CI=-0.3749 – -0.0302, p=0.0213

**Abbreviations:**

AP=antipsychotic drug, PBO=placebo

**Figure S3 Meta-regression analysis in adjunctive therapy**

**(A) Association between treatment response and mean age**


Coefficient=-0.0249, 95% CI=-0.0468 – -0.0029, p=0.0265

**(B) Association between treatment response and number of lifetime depressive episodes**

Coefficient=0.1090, 95% CI=0.0070 – 0.2110, p=0.0363

**(C) Association between discontinuation due to adverse event and sample size**

Coefficient=0.0014, 95% CI=0.0002 – 0.0025, p=0.0170

**(D) Association between discontinuation due to adverse event and DDD ratio**

Coefficient=1.7567, 95% CI=0.8129 – 2.7004, p=0.0003

**Abbreviations:**

AE=adverse event, AP=antipsychotic drug, DDD=daily defined dose, PBO=placebo

**Figure S4 Publication bias assessed by funnel plots, fail-safe estimates and Egger’s tests for the co-primary outcomes**

Note that comparisons with less than three published studies could not be assessed by these methods.

1. **Treatment response, monotherapy; all RCTs**

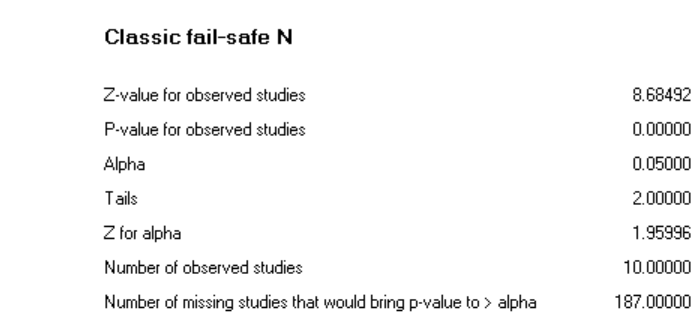


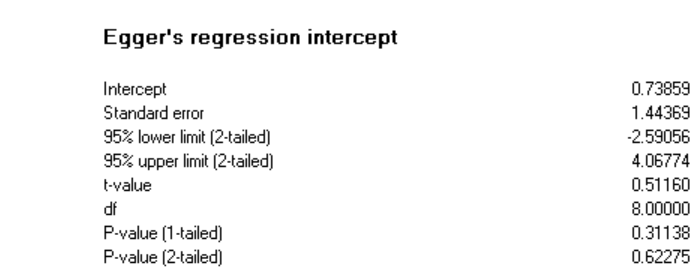


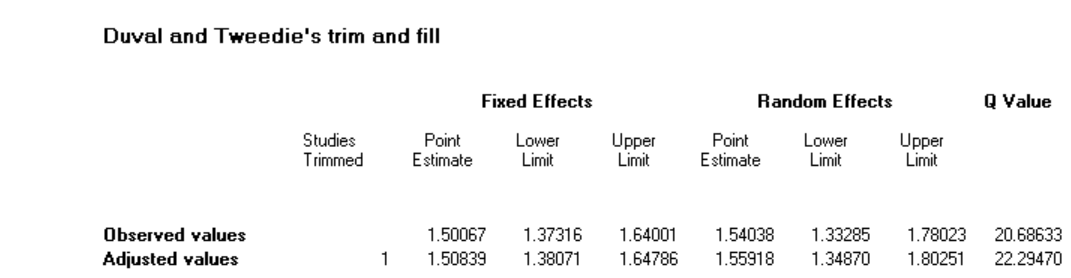


1. **Treatment response, monotherapy; RCTs of QUE**

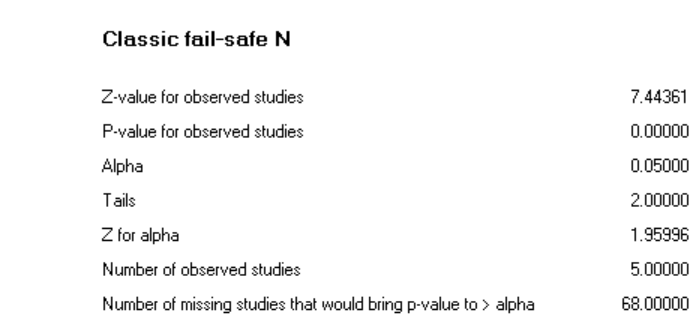


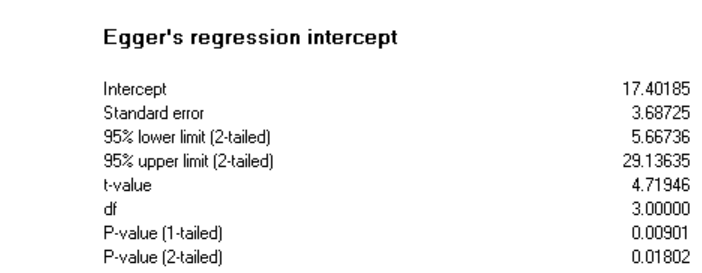


1. **Discontinuation due to adverse event, monotherapy; all RCTs**

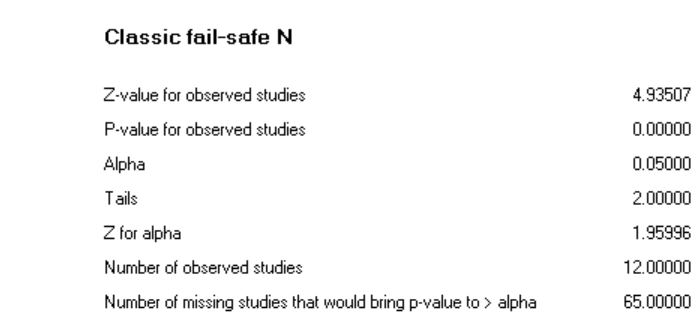


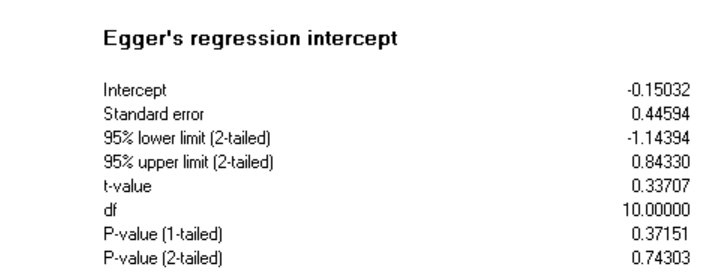


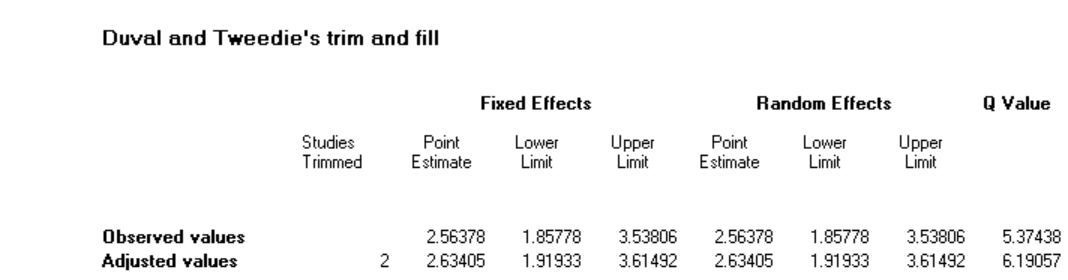


1. **Discontinuation due to adverse event, monotherapy; RCTs of QUE**

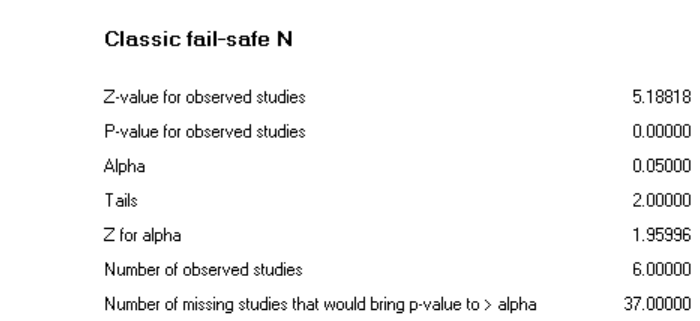


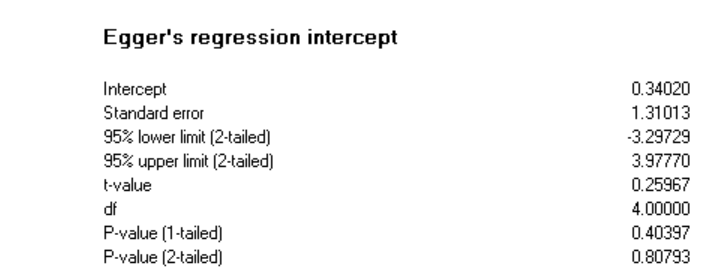


1. **Treatment response, adjunctive therapy; all RCTs**

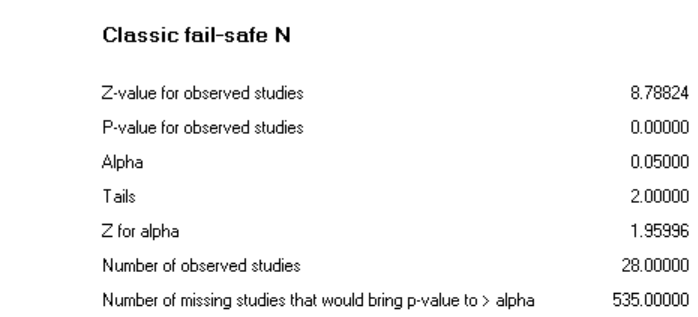


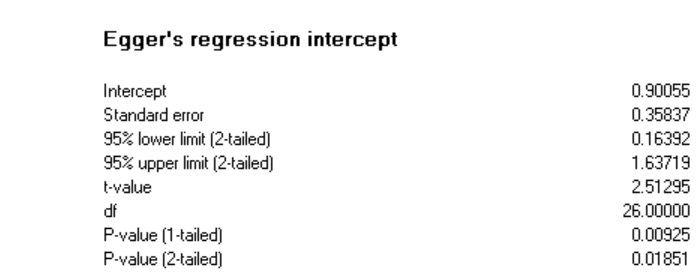


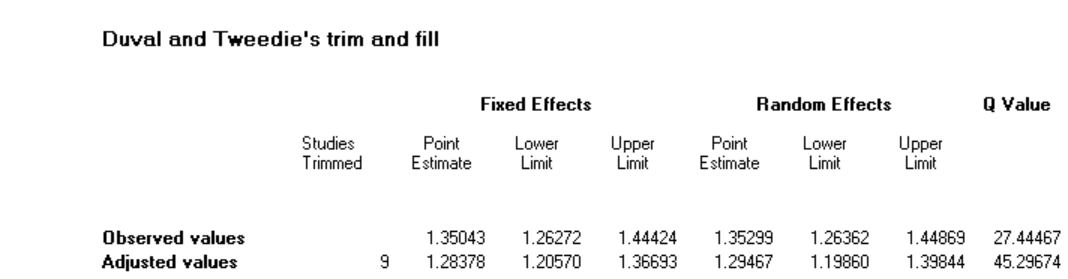


1. **Treatment response, adjunctive therapy; RCTs of ARI**

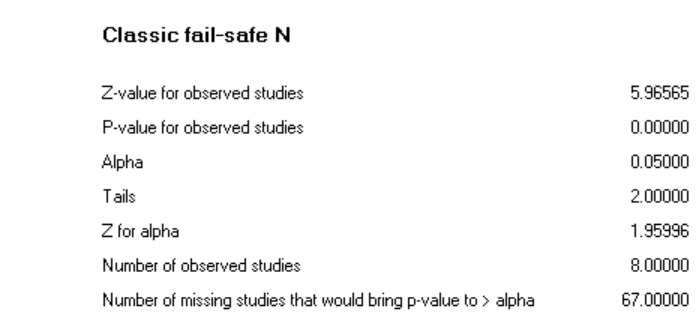


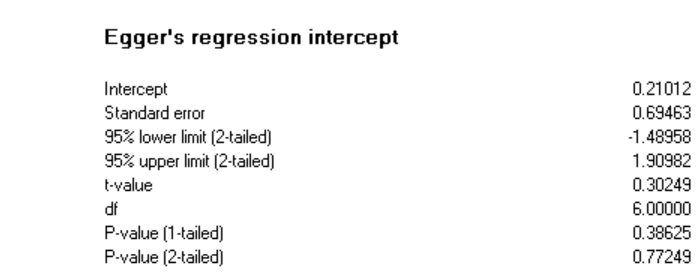


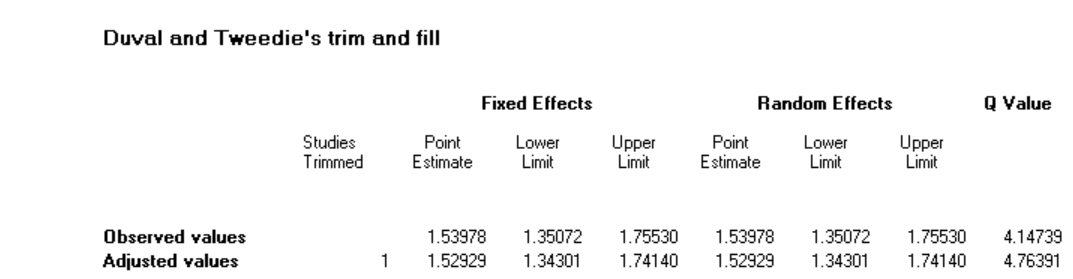


1. **Treatment response, adjunctive therapy; RCTs of BRE**

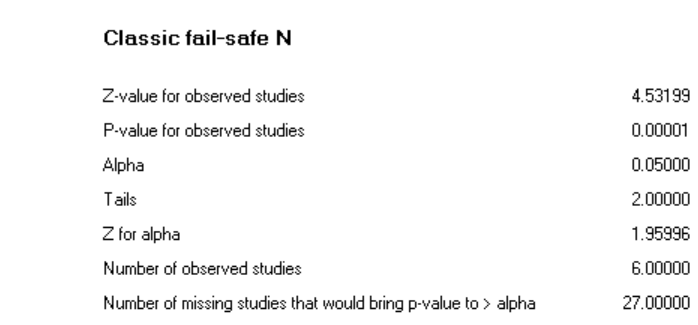


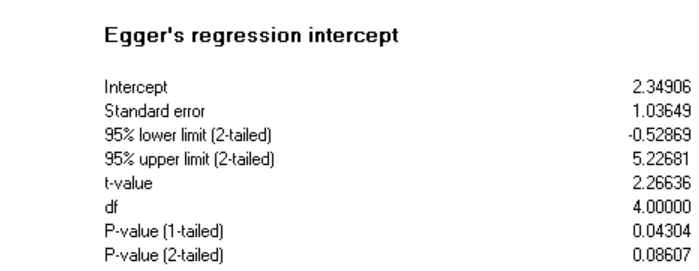


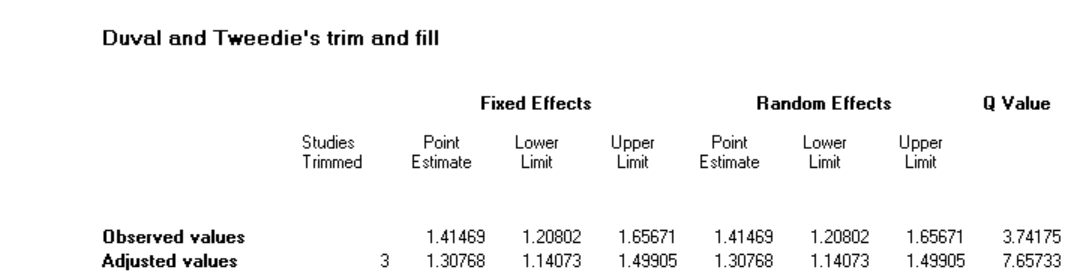


1. **Treatment response, adjunctive therapy; RCTs of QUE**

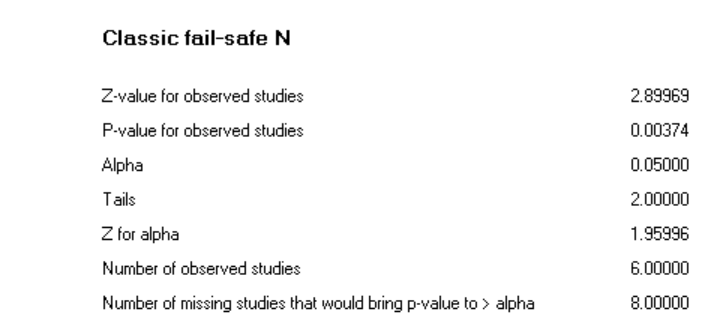


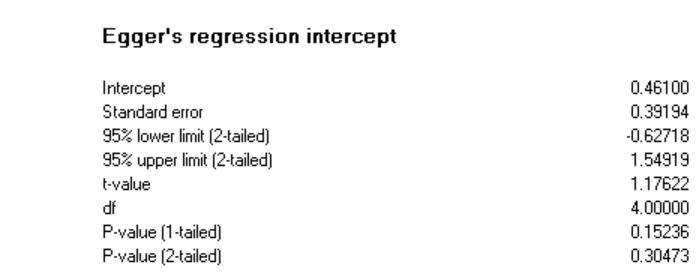


1. **Discontinuation due to adverse event, adjunctive therapy; all RCTs**

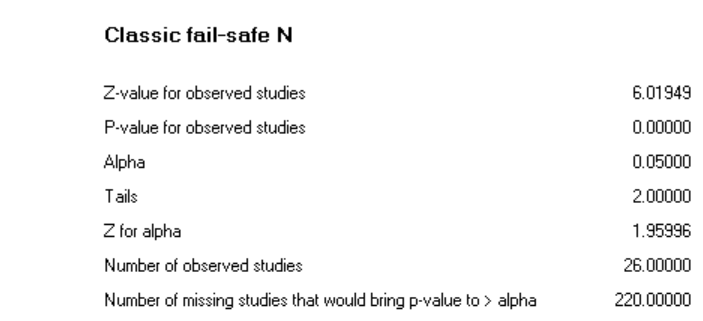


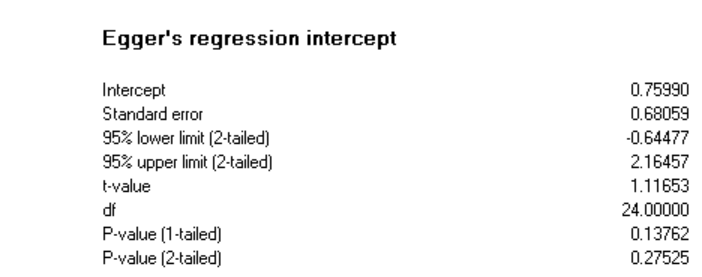


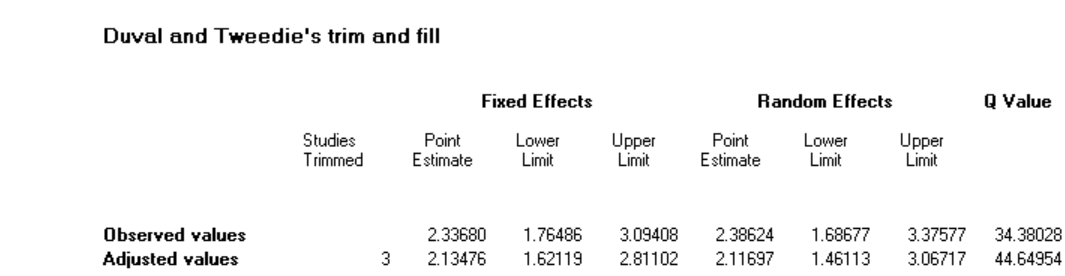


1. **Discontinuation due to adverse event, adjunctive therapy; RCTs of ARI**

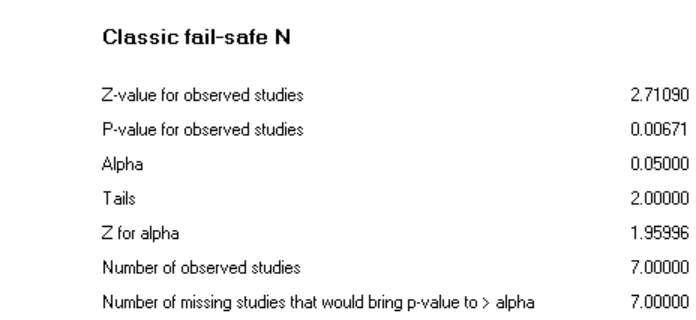


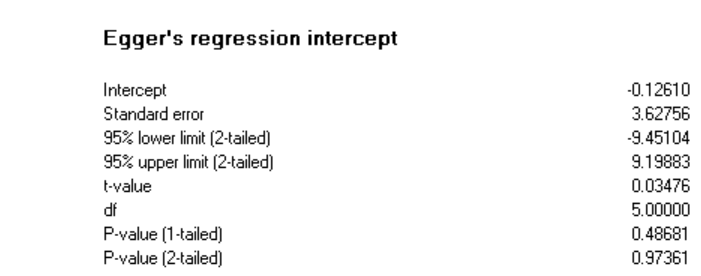


1. **Discontinuation due to adverse event, adjunctive therapy; RCTs of BRE**

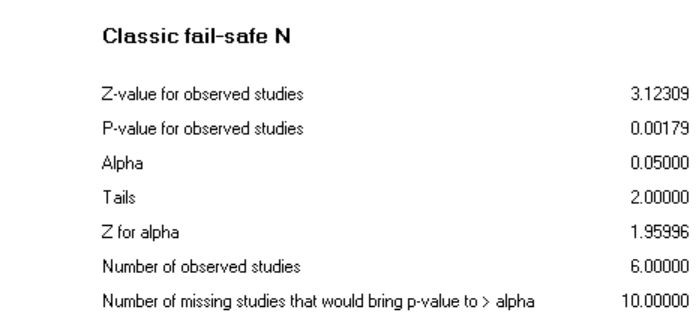


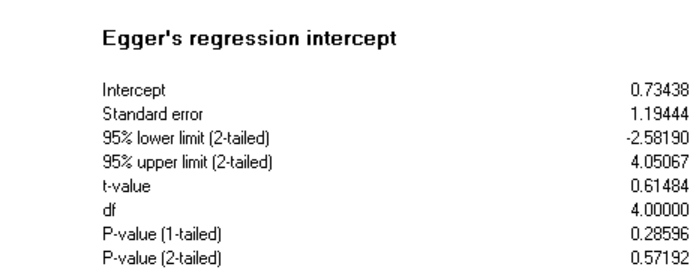


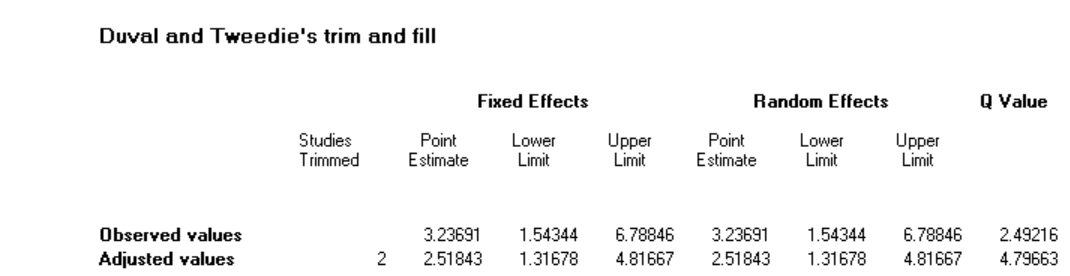


1. **Discontinuation due to adverse event, adjunctive therapy; RCTs of QUE**

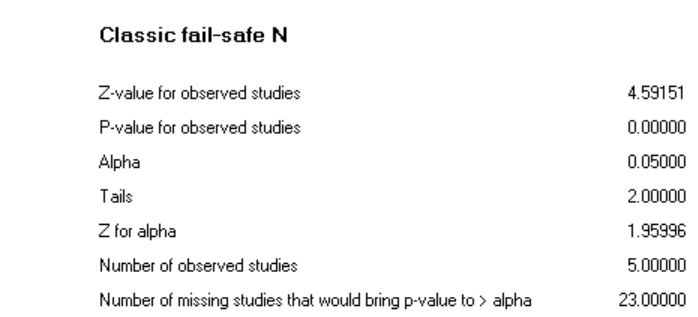


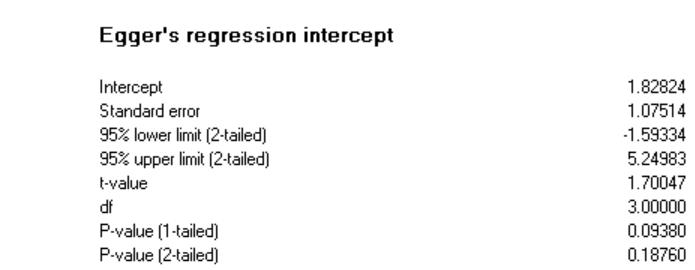


**References**

1. Bortnick B, El-Khalili N, Banov M, Adson D, Datto C, Raines S, et al. Efficacy and tolerability of extended release quetiapine fumarate (quetiapine XR) monotherapy in major depressive disorder: a placebo-controlled, randomized study. Journal of affective disorders. 2011;128(1-2):83-94.

2. Boyer P, Lecrubier Y, Stalla-Bourdillon A, Fleurot O. Amisulpride versus amineptine and placebo for the treatment of dysthymia. Neuropsychobiology. 1999;39(1):25-32.

3. Chaput Y, Magnan A, Gendron A. The co-administration of quetiapine or placebo to cognitive-behavior therapy in treatment refractory depression: a preliminary trial. BMC psychiatry. 2008;8:73.

4. Cutler AJ, Montgomery SA, Feifel D, Lazarus A, Astrom M, Brecher M. Extended release quetiapine fumarate monotherapy in major depressive disorder: a placebo- and duloxetine-controlled study. The Journal of clinical psychiatry. 2009;70(4):526-39.

5. Frolund F. Treatment of depression in general parctice: a controlled trial of flupenthixol ('Fluanxol'). Current medical research and opinion. 1974;2(2):78-89.

6. Katila H, Mezhebovsky I, Mulroy A, Berggren L, Eriksson H, Earley W, et al. Randomized, double-blind study of the efficacy and tolerability of extended release quetiapine fumarate (quetiapine XR) monotherapy in elderly patients with major depressive disorder. The American journal of geriatric psychiatry : official journal of the American Association for Geriatric Psychiatry. 2013;21(8):769-84.

7. Kennedy SH, Giacobbe P, Placenza F, Hudson CJ, Seeman P, Seeman MV. Depression treatment by withdrawal of short-term low-dose antipsychotic, a proof-of-concept randomized double-blind study. Journal of affective disorders. 2014;166:139-43.

8. Lecrubier Y, Boyer P, Turjanski S, Rein W. Amisulpride versus imipramine and placebo in dysthymia and major depression. Amisulpride Study Group. Journal of affective disorders. 1997;43(2):95-103.

9. Liebowitz M, Lam RW, Lepola U, Datto C, Sweitzer D, Eriksson H. Efficacy and tolerability of extended release quetiapine fumarate monotherapy as maintenance treatment of major depressive disorder: a randomized, placebo-controlled trial. Depression and anxiety. 2010;27(10):964-76.

10. Papakostas GI, Vitolo OV, Ishak WW, Rapaport MH, Zajecka JM, Kinrys G, et al. A 12-week, randomized, double-blind, placebo-controlled, sequential parallel comparison trial of ziprasidone as monotherapy for major depressive disorder. The Journal of clinical psychiatry. 2012;73(12):1541-7.

11. Ruther E, Degner D, Munzel U, Brunner E, Lenhard G, Biehl J, et al. Antidepressant action of sulpiride. Results of a placebo-controlled double-blind trial. Pharmacopsychiatry. 1999;32(4):127-35.

12. Wang G, McIntyre A, Earley WR, Raines SR, Eriksson H. A randomized, double-blind study of the efficacy and tolerability of extended-release quetiapine fumarate (quetiapine XR) monotherapy in patients with major depressive disorder. Neuropsychiatric disease and treatment. 2014;10:201-16.

13. Weisler R, Joyce M, McGill L, Lazarus A, Szamosi J, Eriksson H. Extended release quetiapine fumarate monotherapy for major depressive disorder: results of a double-blind, randomized, placebo-controlled study. CNS spectrums. 2009;14(6):299-313.

14. Bauer M, Pretorius HW, Constant EL, Earley WR, Szamosi J, Brecher M. Extended-release quetiapine as adjunct to an antidepressant in patients with major depressive disorder: results of a randomized, placebo-controlled, double-blind study. The Journal of clinical psychiatry. 2009;70(4):540-9.

15. Berman RM, Marcus RN, Swanink R, McQuade RD, Carson WH, Corey-Lisle PK, et al. The efficacy and safety of aripiprazole as adjunctive therapy in major depressive disorder: a multicenter, randomized, double-blind, placebo-controlled study. The Journal of clinical psychiatry. 2007;68(6):843-53.

16. Berman RM, Fava M, Thase ME, Trivedi MH, Swanink R, McQuade RD, et al. Aripiprazole augmentation in major depressive disorder: a double-blind, placebo-controlled study in patients with inadequate response to antidepressants. CNS spectrums. 2009;14(4):197-206.

17. Dunner DL, Amsterdam JD, Shelton RC, Loebel A, Romano SJ. Efficacy and tolerability of adjunctive ziprasidone in treatment-resistant depression: a randomized, open-label, pilot study. The Journal of clinical psychiatry. 2007;68(7):1071-7.

18. Durgam S, Earley W, Guo H, Li D, Nemeth G, Laszlovszky I, et al. Efficacy and safety of adjunctive cariprazine in inadequate responders to antidepressants: a randomized, double-blind, placebo-controlled study in adult patients with major depressive disorder. The Journal of clinical psychiatry. 2016;77(3):371-8.

19. El-Khalili N, Joyce M, Atkinson S, Buynak RJ, Datto C, Lindgren P, et al. Extended-release quetiapine fumarate (quetiapine XR) as adjunctive therapy in major depressive disorder (MDD) in patients with an inadequate response to ongoing antidepressant treatment: a multicentre, randomized, double-blind, placebo-controlled study. The international journal of neuropsychopharmacology. 2010;13(7):917-32.

20. Fava M, Mischoulon D, Iosifescu D, Witte J, Pencina M, Flynn M, et al. A double-blind, placebo-controlled study of aripiprazole adjunctive to antidepressant therapy among depressed outpatients with inadequate response to prior antidepressant therapy (ADAPT-A Study). Psychotherapy and psychosomatics. 2012;81(2):87-97.

21. Flint AJ, Meyers BS, Rothschild AJ, Whyte EM, Alexopoulos GS, Rudorfer MV, et al. Effect of Continuing Olanzapine vs Placebo on Relapse Among Patients With Psychotic Depression in Remission: The STOP-PD II Randomized Clinical Trial. Jama. 2019;322(7):622-31.

22. Garakani A, Martinez JM, Marcus S, Weaver J, Rickels K, Fava M, et al. A randomized, double-blind, and placebo-controlled trial of quetiapine augmentation of fluoxetine in major depressive disorder. International clinical psychopharmacology. 2008;23(5):269-75.

23. Hobart M, Skuban A, Zhang P, Augustine C, Brewer C, Hefting N, et al. A Randomized, Placebo-Controlled Study of the Efficacy and Safety of Fixed-Dose Brexpiprazole 2 mg/d as Adjunctive Treatment of Adults With Major Depressive Disorder. The Journal of clinical psychiatry. 2018;79(4).

24. Hobart M, Skuban A, Zhang P, Josiassen MK, Hefting N, Augustine C, et al. Efficacy and safety of flexibly dosed brexpiprazole for the adjunctive treatment of major depressive disorder: a randomized, active-referenced, placebo-controlled study. Current medical research and opinion. 2018;34(4):633-42.

25. Ionescu DF, Fava M, Kim DJ, Baer L, Shelton RC, Cusin C. A placebo-controlled crossover study of iloperidone augmentation for residual anger and irritability in major depressive disorder. Therapeutic advances in psychopharmacology. 2016;6(1):4-12.

26. Kamijima K, Higuchi T, Ishigooka J, Ohmori T, Ozaki N, Kanba S, et al. Aripiprazole augmentation to antidepressant therapy in Japanese patients with major depressive disorder: a randomized, double-blind, placebo-controlled study (ADMIRE study). Journal of affective disorders. 2013;151(3):899-905.

27. Kamijima K, Kimura M, Kuwahara K, Kitayama Y, Tadori Y. Randomized, double-blind comparison of aripiprazole/sertraline combination and placebo/sertraline combination in patients with major depressive disorder. Psychiatry and clinical neurosciences. 2018;72(8):591-601.

28. Keitner GI, Garlow SJ, Ryan CE, Ninan PT, Solomon DA, Nemeroff CB, et al. A randomized, placebo-controlled trial of risperidone augmentation for patients with difficult-to-treat unipolar, non-psychotic major depression. Journal of psychiatric research. 2009;43(3):205-14.

29. Lenze EJ, Mulsant BH, Blumberger DM, Karp JF, Newcomer JW, Anderson SJ, et al. Efficacy, safety, and tolerability of augmentation pharmacotherapy with aripiprazole for treatment-resistant depression in late life: a randomised, double-blind, placebo-controlled trial. Lancet (London, England). 2015;386(10011):2404-12.

30. Li R, Wu R, Chen J, Kemp DE, Ren M, Conroy C, et al. A Randomized, Placebo-Controlled Pilot Study of Quetiapine-XR Monotherapy or Adjunctive Therapy to Antidepressant in Acute Major Depressive Disorder with Current Generalized Anxiety Disorder. Psychopharmacology bulletin. 2016;46(1):8-23.

31. Lin CH, Lin SH, Jang FL. Adjunctive low-dose aripiprazole with standard-dose sertraline in treating fresh major depressive disorder: a randomized, double-blind, controlled study. Journal of clinical psychopharmacology. 2011;31(5):563-8.

32. Mahmoud RA, Pandina GJ, Turkoz I, Kosik-Gonzalez C, Canuso CM, Kujawa MJ, et al. Risperidone for treatment-refractory major depressive disorder: a randomized trial. Annals of internal medicine. 2007;147(9):593-602.

33. Marcus RN, McQuade RD, Carson WH, Hennicken D, Fava M, Simon JS, et al. The efficacy and safety of aripiprazole as adjunctive therapy in major depressive disorder: a second multicenter, randomized, double-blind, placebo-controlled study. Journal of clinical psychopharmacology. 2008;28(2):156-65.

34. McIntyre A, Gendron A, McIntyre A. Quetiapine adjunct to selective serotonin reuptake inhibitors or venlafaxine in patients with major depression, comorbid anxiety, and residual depressive symptoms: a randomized, placebo-controlled pilot study. Depression and anxiety. 2007;24(7):487-94.

35. Papakostas GI, Fava M, Baer L, Swee MB, Jaeger A, Bobo WV, et al. Ziprasidone Augmentation of Escitalopram for Major Depressive Disorder: Efficacy Results From a Randomized, Double-Blind, Placebo-Controlled Study. The American journal of psychiatry. 2015;172(12):1251-8.

36. Parker G, Brotchie H, Parker K. Is combination olanzapine and antidepressant medication associated with a more rapid response trajectory than antidepressant alone? The American journal of psychiatry. 2005;162(4):796-8.

37. Quante A, Regen F, Schindler F, Volkmer K, Severus E, Urbanek C, et al. Quetiapine as combination treatment with citalopram in unipolar depression with prominent somatic symptoms: a randomised, double-blind, placebo-controlled pilot study. Psychiatria Danubina. 2013;25(3):214-20.

38. Reeves H, Batra S, May RS, Zhang R, Dahl DC, Li X. Efficacy of risperidone augmentation to antidepressants in the management of suicidality in major depressive disorder: a randomized, double-blind, placebo-controlled pilot study. The Journal of clinical psychiatry. 2008;69(8):1228-36.

39. Sim M, Gordon EB, Nicol CG. Oxypertine in combination with imipramine: a controlled trial. The Journal of international medical research. 1978;6(1):4-10.

40. Stabl M, Kasas A, Blajev B, Bajetta G, Zochling R, Holsboer-Trachsler E, et al. A double-blind comparison of moclobemide and thioridazine versus moclobemide and placebo in the treatment of refractory, severe depression. Journal of clinical psychopharmacology. 1995;15(4 Suppl 2):41s-5s.

41. Thase ME, Youakim JM, Skuban A, Hobart M, Augustine C, Zhang P, et al. Efficacy and safety of adjunctive brexpiprazole 2 mg in major depressive disorder: a phase 3, randomized, placebo-controlled study in patients with inadequate response to antidepressants. The Journal of clinical psychiatry. 2015;76(9):1224-31.

42. Thase ME, Youakim JM, Skuban A, Hobart M, Zhang P, McQuade RD, et al. Adjunctive brexpiprazole 1 and 3 mg for patients with major depressive disorder following inadequate response to antidepressants: a phase 3, randomized, double-blind study. The Journal of clinical psychiatry. 2015;76(9):1232-40.

43. Wade AG, Crawford GM, Nemeroff CB, Schatzberg AF, Schlaepfer T, McConnachie A, et al. Citalopram plus low-dose pipamperone versus citalopram plus placebo in patients with major depressive disorder: an 8-week, double-blind, randomized study on magnitude and timing of clinical response. Psychological medicine. 2011;41(10):2089-97.

44. Study of the Safety and Efficacy of OPC-34712 as Adjunctive Therapy in the Treatment of Patients With Major Depressive Disorder [Available from: <https://clinicaltrials.gov/ct2/show/NCT00797966>.

45. Study of the Safety and Efficacy of OPC-34712 as Adjunctive Therapy in the Treatment of Adults With Major Depressive Disorder (STEP-D222) [Available from: <https://clinicaltrials.gov/ct2/show/NCT01052077>.
